# Mediation analysis methods used in observational research: a scoping review and recommendations

**DOI:** 10.1186/s12874-021-01426-3

**Published:** 2021-10-25

**Authors:** Judith J. M. Rijnhart, Sophia J. Lamp, Matthew J. Valente, David P. MacKinnon, Jos W. R. Twisk, Martijn W. Heymans

**Affiliations:** 1 Department of Epidemiology and Data Science, Amsterdam UMC, Location VU University Medical Center, Amsterdam Public Health Research Institute, PO Box 7057, 1007 MB Amsterdam, The Netherlands; 2 Department of Psychology, Arizona State University, Tempe, AZ USA; 3 Department of Psychology, Center for Children and Families, Florida International University, Miami, FL USA

**Keywords:** Mediation analysis, Counterfactuals, Potential outcomes, Indirect effect, Direct effect, Observational data

## Abstract

**Background:**

Mediation analysis methodology underwent many advancements throughout the years, with the most recent and important advancement being the development of causal mediation analysis based on the counterfactual framework. However, a previous review showed that for experimental studies the uptake of causal mediation analysis remains low. The aim of this paper is to review the methodological characteristics of mediation analyses performed in observational epidemiologic studies published between 2015 and 2019 and to provide recommendations for the application of mediation analysis in future studies.

**Methods:**

We searched the MEDLINE and EMBASE databases for observational epidemiologic studies published between 2015 and 2019 in which mediation analysis was applied as one of the primary analysis methods. Information was extracted on the characteristics of the mediation model and the applied mediation analysis method.

**Results:**

We included 174 studies, most of which applied traditional mediation analysis methods (*n* = 123, 70.7%). Causal mediation analysis was not often used to analyze more complicated mediation models, such as multiple mediator models. Most studies adjusted their analyses for measured confounders, but did not perform sensitivity analyses for unmeasured confounders and did not assess the presence of an exposure-mediator interaction.

**Conclusions:**

To ensure a causal interpretation of the effect estimates in the mediation model, we recommend that researchers use causal mediation analysis and assess the plausibility of the causal assumptions. The uptake of causal mediation analysis can be enhanced through tutorial papers that demonstrate the application of causal mediation analysis, and through the development of software packages that facilitate the causal mediation analysis of relatively complicated mediation models.

**Supplementary Information:**

The online version contains supplementary material available at 10.1186/s12874-021-01426-3.

## Background

Mediation analysis is increasingly being applied in many research fields [1], including the field of epidemiology. Mediation analysis decomposes the total exposure-outcome effect into a direct effect and an indirect effect through a mediator variable [2–4]. For example, mediation analysis can be used to investigate BMI as a mediator of the relation between smoking and insulin levels [5], or to investigate food expenditures as a mediator of the relation between socioeconomic status and healthiness of food choices [6]. Mediation analysis is therefore an important statistical tool for gaining insight into the mechanisms of exposure-outcome effects [3].

Throughout the years, various methods for mediation analysis have been described in the literature. Building on the path analysis method described by Sewall Wright [7, 8], Judd and Kenny described the causal steps method in 1981 [9], followed by an adaptation of this method in 1986 by Baron and Kenny [10]. The causal steps method relies on a sequence of significance tests to determine the presence of a mediated effect. Later papers recommended estimating the indirect effect based on the product-of-coefficients method or the difference-in-coefficients method to determine the presence of a mediated effect [3, 11–13]. Here we refer to these methods as ‘traditional mediation analysis’. In the last decade, causal mediation analysis gained popularity. Causal mediation analysis provides general definitions of causal direct, indirect, and total effects, which can be estimated using various estimation approaches [4, 14, 15]. Causal and traditional mediation analysis can provide the same effect estimates for mediation models estimated with linear regression [16, 17], but this does not necessarily hold for mediation models estimated with non-linear regression [18, 19]. Causal mediation analysis is preferred for the latter models, as for these models causal mediation analysis provides causal effect estimates, while traditional mediation analysis can in some situations only be used to test the presence of a mediated effect [19].

Although the theoretical definitions of the causal direct, indirect, and total effects are not new [4, 14, 15], the uptake of causal mediation analysis in practice has remained low for many years [20]. In the past decade, various software programs have been developed for the estimation of causal mediation effects, enabling researchers to perform causal mediation analysis in all major software packages (i.e., SAS, SPSS, Stata, R, and M*plus*) [21–28]. However, it is not clear whether these software packages increased the uptake of causal mediation analysis in epidemiologic research. A recent review showed that traditional mediation analysis is still most frequently used to analyze data from randomized controlled trials [29]. It remains unclear whether this also holds for observational studies, which are common in the field of epidemiologic research.

## Methods

### Aim

The aim of this paper is to review the methodological characteristics of mediation analyses performed in observational epidemiologic studies published between 2015 and 2019 and to provide recommendations for the application of mediation analyses in future studies. In this paper we performed a scoping review, as the aim of this paper is relatively broad and concerns the collection of information on a range of methodological characteristics rather than information on a clearly defined substantive question [30]. In the next section, we first provide an overview of traditional and causal mediation analysis methods. Then we describe the methods and results of our scoping review. Finally, we provide recommendations for the application of mediation analysis in future studies.

### Traditional mediation analysis

**Fig. 1:**
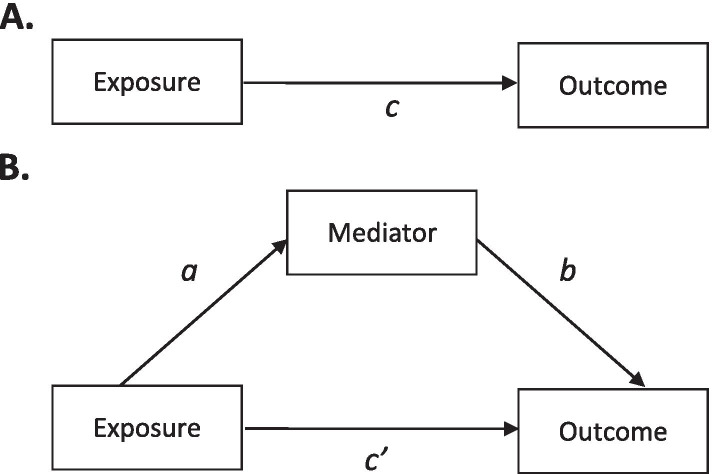
Path diagram of a single mediator model.

Traditional mediation analysis is based on the estimation of the four pathways shown in Fig. 1 [3, 10]. In Fig. 1A, the *c* path represents the total exposure-outcome effect. In Fig. 1B, the *a* path represents the exposure-mediator effect, the *b* path represents the mediator-outcome effect, and the *c’* path represents the direct exposure-outcome effect. When the mediator and outcome are both continuous, the paths in Fig. 1 are estimated using the following three linear regression eqs. (9):

1 $$Y={i}_1+ cX+{d}_1Z+{\upvarepsilon}_1$$

2 $$M={i}_2+ aX+{d}_2Z+{\upvarepsilon}_2$$

3 $$Y={i}_3+{c}^{\prime }X+ bM+{d}_3Z+{\upvarepsilon}_3$$

where the *c* coefficient in eq. 1 represents the total exposure-outcome effect. The *a* coefficient in eq. 2 represents the exposure-mediator effect. The *b* coefficient in eq. 3 represents the mediator-outcome effect when adjusted for the exposure, and the *c’* coefficient represents the direct exposure-outcome effect when adjusted for the mediator. The *i*_*1*_, *i*_*2*_, and *i*_*3*_ terms represent intercepts and the *ε*_*1*_, *ε*_*2*_, and *ε*_*3*_ terms represent residuals. Finally, *Z* represents a set of confounders. The inclusion of confounders in eqs. 1, 2, and 3 should always be considered when a mediation analysis is performed based on observational data, as the exclusion of confounders will result in biased effect estimates [3].

Traditional mediation analysis defines the direct, indirect, and total effects in terms of the linear regression coefficients from eqs. 1, 2, and 3 [3, 12]. The total effect is defined and estimated as the *c* coefficient from eq. 1 and the direct effect is defined and estimated as the *c’* coefficient from eq. 3. The indirect effect is defined and estimated as the product of the *a* and *b* coefficients (*ab*) and as the difference between the *c* coefficient and the *c’* coefficient (*c-c’*). These two indirect effects are mathematically equivalent when the regression coefficients are estimated with linear regression [13]. The relative size of the mediated effect can be assessed using the proportion mediated, which represents the size of the indirect effect estimate relative to the total effect estimate, or by interpreting the standardized indirect effect estimate as a Cohen’s *d* [3].

Some of the first papers on mediation analysis recommended to assess the statistical significance of the indirect effect estimate with a *z*-test or a confidence interval based on the multivariate delta standard error [10, 31–33]. However, these methods are not recommended, as they assume that the indirect effect estimate follows a normal sampling distribution, which often does not hold [34]. As a result, the *z*-test and confidence interval based on the multivariate delta standard error have relatively low power to detect a statistically significant indirect effect [35–37]. Confidence intervals that do take into account the nonnormal sampling distribution of the indirect effect estimator are therefore preferred, such as the distribution of the product confidence interval, Monte Carlo confidence interval, and bootstrap confidence intervals [34, 36, 38].

Mediation analysis is based on the assumption of temporal precedence of the exposure, mediator, and outcome, which means that changes in the exposure are assumed to precede changes in the mediator, and that changes in the mediator are assumed to precede changes in the outcome [3, 39]. Furthermore, traditional mediation analysis is based on parametric regression assumptions. In other words, the residuals of the linear regression models are assumed to be normally distributed and homoscedastic across values of the independent variables in the model, the *a*, *b*, *c*, and *c’* coefficients are assumed to represent their correct functional form (e.g., linear or quadratic), the observations are assumed to be independent, and it is assumed that there are no effect modifiers or omitted confounders of the estimated effects [3, 40]. Effect modifiers can be taken into account by including interaction terms (i.e., exposure-by-covariate or mediator-by-covariate) in the models and by subsequently estimating the direct and indirect effects for different values of the effect modifier. This can, for example, be done by estimating the effects for specific categories of a categorical effect modifier or by centering a continuous effect modifier at a clinically relevant valu*e* [3, 11]. The effect estimates can be adjusted for measured confounders by adding the confounder variables to all estimated regression equations.

Ambiguities arise when traditional mediation analysis is used to estimate the effects for mediation models with non-continuous mediator and outcome variables [12, 41, 42]. For example, the product-of coefficients and difference-in-coefficients methods provide different indirect effect estimates when based on the coefficients from non-linear regression models, such as logistic regression or Cox proportional-hazards regression [12, 41, 43]. Furthermore, although it has been recommended to assess the presence of exposure-mediator interactions in the traditional mediation analysis literature, guidance is scarce on the estimation and interpretation of effects for mediation models with an exposure-mediator interaction [3, 9]. Recent papers have shown that group-mean centering of the continuous mediator variable in traditional mediation analysis yields effect estimates similar to the effect estimates from causal mediation analysis for mediation models with a continuous outcome and an exposure-mediator interaction [16], but not necessarily for mediation models with a binary outcome and an exposure-mediator interaction [18].

### Causal mediation analysis

Causal mediation analysis clarifies the ambiguities that arise in traditional mediation analysis [16, 18, 44]. Causal mediation analysis is based on the counterfactual framework [4, 14, 15], and distinguishes causal effect definitions from causal effect estimation [45]. A strength of the causal effect definitions is that they are non-parametric and therefore can be applied to any type of mediation model to derive the causal effect estimates. This includes models with an exposure-mediator interaction and models with non-continuous mediator variables or non-continuous outcome variables [46].

#### Causal effect definitions

Causal mediation analysis defines causal effects as the difference between two counterfactual outcomes [47, 48]. A counterfactual outcome is an individual’s outcome value that would be observed when exposed to a certain exposure value. In the remainder of this section we denote the outcome as Y, and the exposure values of interest as *x* and x*. In theory, two counterfactual outcomes can be observed for one individual over the same time period, one based on exposure value *x* and one based on exposure value x* [47, 48]. The individual’s counterfactual outcome under exposure value *x* is denoted as Y_i_(*x*), and the individual’s counterfactual outcome under exposure value x* is denoted as Y_i_(x*). The causal exposure effect is defined as the difference between these two counterfactual outcomes observed for the same individual over the same time period, i.e., *Y*_*i*_(*x*) – *Y*_*i*_(*x*^∗^).

The counterfactual outcomes in a mediation model are not only dependent on exposure values, but also on mediator values [4]. We denote the mediator as M and the mediator values as *m*. The counterfactual notation for the outcome can be extended by including this mediator value. An individual’s counterfactual outcome under exposure value *x* and mediator value *m* is denoted as Y_i_(*x*, *m*), and the same individual’s counterfactual outcome under exposure value x* and mediator value *m* as Y_i_(x*, *m*). The difference between these two counterfactual outcomes observed for the same individual over the same time period is the controlled direct effect (CDE), i.e., *Y*_*i*_(*x*, *m*) – *Y*_*i*_(*x*^∗^, *m*). The CDE is the direct effect of changing an individual’s exposure value from *x* to x*, while holding the mediator value constant at *m* [4]. The mediator value *m* is determined by the researcher and reflects a value of clinical or policy relevance [4].

Instead of holding the mediator constant at a predetermined value, we can also let the mediator take on the value that would naturally be observed under exposure values *x* and x* [4]. Two counterfactual mediator values can be observed for an individual under the two exposure values *x* and x*: the counterfactual mediator value under exposure value *x*, i.e., M_i_(*x*), and the counterfactual mediator value under exposure value x*, i.e., M_i_(x*). We can now replace mediator value *m* with these two counterfactual mediator values, resulting in four nested counterfactual outcome values: *Y*_*i*_(*x*, *M*_*i*_(*x*)), *Y*_*i*_(*x*, *M*_*i*_(*x*^∗^)), *Y*_*i*_(*x*^∗^, *M*_*i*_(*x*)), and *Y*_*i*_(*x*^∗^, *M*_*i*_(*x*^∗^)) [4, 49]. These four counterfactual outcomes are referred to as nested counterfactual outcomes, because the counterfactual mediator values are nested within the counterfactual outcomes values [4].

Five causal effects are defined based on the differences between these nested counterfactual outcomes: the pure natural direct effect (PNDE), the total natural direct effect (TNDE), the pure natural indirect effect (PNIE), the total natural indirect effect (TNIE), and the total effect (TE) [4, 15]. Table 1 provides an overview of these causal effects and their respective interpretations. For the natural direct effects we block the effect through the mediator by holding each individual’s mediator constant at either M_i_(*x*) or M_i_(x*), while for the natural indirect effects we block the effect through the exposure by holding the exposure constant at either *x* and x* [1, 50]. For the TE, we allow information to flow through both the exposure and mediator, varying both the exposure value and the counterfactual mediator value.

**Table 1:** Overview of the definitions and interpretations of the causal mediation effects

Effect	Definition	Interpretation
CDE	*Y*_*i*_(*x*, *m*) – *Y*_*i*_(*x*^∗^, *m*)	The direct effect of changing the individual’s exposure value from *x* to x*, while holding the mediator value constant at *m.*
PNDE	*Y*_*i*_(*x*, *M*_*i*_(*x*^∗^)) − *Y*_*i*_(*x*^∗^, *M*_*i*_(*x*^∗^))	The natural direct effect of changing the individual’s exposure value from *x* to x*, while holding the mediator for that individual constant at the value that would naturally be observed under exposure value x*.
TNDE	*Y*_*i*_(*x*, *M*_*i*_(*x*)) − *Y*_*i*_(*x*^∗^, *M*_*i*_(*x*))	The natural direct effect of changing the individual’s exposure value from *x* to x*, while holding the mediator for that individual constant at the value that would naturally be observed under exposure value *x.*
PNIE	*Y*_*i*_(*x*^∗^, *M*_*i*_(*x*)) − *Y*_*i*_(*x*^∗^, *M*_*i*_(*x*^∗^))	The natural indirect effect of changing the individual’s mediator value from M(x*) to M(*x*), while holding the individual’s exposure value constant at x*.
TNIE	*Y*_*i*_(*x*, *M*_*i*_(*x*)) − *Y*_*i*_(*x*, *M*_*i*_(*x*^∗^))	The natural indirect effect of changing the individual’s mediator value from M(x*) to M(*x*), while holding the individual’s exposure value constant at *x.*
TE	*Y*_*i*_(*x*, *M*_*i*_(*x*)) − *Y*_*i*_(*x*^∗^, *M*_*i*_(*x*^∗^))	The total effect of changing the individual’s exposure value from *x* to x*.

*Abbreviations:* CDE, controlled direct effect; PNDE, pure natural direct effect; TNDE, total natural direct effect; PNIE, pure natural indirect effect; TNIE, total natural indirect effect; TE, total effect

The causal effects are defined at the individual level, but in practice we are unable to observe multiple counterfactual outcomes for the same individual over the same time period [47, 48]. Therefore, we are unable to estimate individual-level causal effects. This has been referred to as the *fundamental problem of causal inference* [47]. Instead, we can estimate the population-average causal effects based on the expected difference between two population-average (nested) counterfactual outcomes [4, 14, 47]. To ensure that the PNDE, TNDE, PNIE, and TNIE have a causal interpretation at the population-average level, the following four assumptions need to hold [4, 46]:

1. no unmeasured confounding of the exposure-outcome effect;
2. no unmeasured confounding of the mediator-outcome effect;
3. no unmeasured confounding of the exposure-mediator effect;
4. no confounders of the mediator-outcome effect that are affected by the exposure.

Assumption 4 is also known as the cross-world independence assumption. In practice this is often a strong assumption [51], for example because often there will be multiple mediators of the exposure-outcome effect. For the CDE only assumptions 1 and 2 have to hold, and for the TE only assumption 1 has to hold. Finally, consistency is assumed, which means that the observed mediator and outcome values would also have been observed had the individual randomly been assigned the observed exposure and mediator values [46, 52].

#### Causal effect estimation

Various estimation approaches have been developed to estimate the causal direct, indirect, and total effects at the population-average level, including simulations, numerical integration, multiple regression analysis, and natural effect models [19, 23, 53–55]. Most of these methods use eq. 2 and/or eq. 3 as input. Provided that the relevant parametric assumptions hold, the regression coefficients from eqs. 2 and 3 can be used to compute the causal mediation effects. To accommodate the estimation of pure and total natural direct and indirect effects, eq. 3 is typically extended with an exposure-mediator interaction term.

The simulation-based approach can be applied based on both parametric and non-parametric models [25, 53]. The parametric simulation-based approach uses the sampling distributions of the estimated parameters from eqs. 2 and 3 to simulate the potential mediator and outcome values for each subject. Based on the simulated potential outcomes, the causal effects are computed for each subject. Subsequently, the causal effects are averaged to arrive at the population-average causal effects. The non-parametric simulation-based approach estimates possibly non-parametric models for the mediator and outcome variables within a prespecified number of bootstrap resamples. Based on these models the potential mediator and outcome values are simulated for each subject. Then based on these simulated potential outcomes, the causal effects are estimated and averaged to get the population-average causal effects.

Numerical integration uses eqs. 2 and 3 as input [4, 23]. Based on these equations, average expected outcome values are estimated conditional on the two exposure levels of interest, i.e., *x* and x*, and all mediator values. These expected outcome values are weighted by the mediator distributions observed under *x* and x* to estimate the population-average nested potential outcomes, which are subsequently subtracted to get the population-average causal effect estimates.

The regression-based method estimates the average potential outcomes based on the regression coefficients in eqs. 2 and 3 [19, 46, 56]. These estimated potential outcomes are subsequently subtracted to estimate the population-average causal mediation effects. The regression-based effects for mediation models with a binary or time-to-event outcome were originally derived on the risk-ratio scale, therefore this method poses an additional rare outcome assumption when the causal effects are estimated on the odds-ratio scale or hazard-ratio scale [56, 57]. This assumption requires the outcome prevalence to be low across all strata of the exposure and mediator variable [58]. When this assumption is violated, the effect estimates on the odds-ratio scale or hazard-ratio scale can still be used to assess the presence of a mediated effect, but they do not have a causal interpretation [56]. To ensure a causal interpretation, the effects can alternatively be estimated on the risk-ratio scale using log-linear regression or on the survival-time ratio scale using accelerated failure time models [28, 57].

In natural effect models the natural direct effect and natural indirect effect are each represented by a single regression coefficient [25]. In contrast with the other estimation methods, natural effect models require the estimation of only one of the aforementioned regression equations, i.e., eqs. 2 and 3, in addition to the natural effect model [59]. Natural effect models are estimated using a weighting-based approach or a imputation-based approach. The weighting-based approach creates an expanded dataset with weights for each subject based on eq. 2 [54, 60]. The natural effects model is subsequently estimated by regressing the outcome on the two exposure values of interested, i.e., *x* and x*, and the covariates, while weighting each observation based on the computed weights. The imputation-based approach creates an expanded dataset in which the missing potential outcome values are imputed based on information from eq. 3 [55]. Based on this complete dataset, a natural effects model is estimated.

### Traditional mediation analysis versus causal mediation analysis

For certain mediation models, traditional mediation analysis provides the same effect estimates as causal mediation analysis. Traditional mediation analysis provides the same effect estimates as causal mediation analysis for single mediator models with a continuous mediator and a continuous outcome [16, 17, 45]. This also means that traditional mediation analysis fails to provide causal effect estimates when the four no (unmeasured) confounding assumptions are violated. For mediation models with a binary or time-to-event outcome, traditional and causal mediation analysis do not necessarily provide the same effect estimates [16, 18]. For these models, the effect estimation in traditional mediation analysis is most closely related to the regression-based estimation approach in causal mediation analysis, which also estimates the indirect effect using the product-of-coefficients method in the absence of exposure-mediator interaction. However, an important difference is the rare outcome assumption posed by causal mediation analysis for mediation models with a binary or time-to-event outcome. This rare outcome assumption clarifies that the traditional effect estimates based on logistic regression and Cox proportional hazards regression only have a causal interpretation when the outcome is rare.

When there are multiple mediators of the exposure-outcome effect, it is important to take into account all these mediators, because they may be correlated or they may influence one another violating the fourth no confounding assumption, i.e., no confounders of the mediator-outcome effect that are affected by the exposure. Causal mediation analysis clarifies the necessary additional causal assumptions for models with multiple mediators and various methods have been developed for the estimation of causal effects for multiple mediator models [25, 61–63].

In recent years, various causal mediation software packages have been developed that enable researchers to apply causal mediation analysis based on only a few lines of code [21–27, 64]. However, it remains unclear whether the availability of these causal mediation programs has increased the uptake of causal mediation analysis in practice. In the next section we describe the set-up of our scoping review in which we collected information on the methodological characteristics of mediation analyses in published observational studies, with a special focus on the mediation analysis method used.

### Study design

This scoping review is reported in accordance with the Preferred Reporting Items for Systematic Reviews and Meta-Analyses (PRISMA) statement [65] and the PRISMA-ScR extension [66]. The PRISMA-ScR checklist can be found in supplementary appendix 1. The protocol for this scoping review was not registered in the international register of systematic reviews, because we did not extract data on clinical outcomes [67].

Our search strategy is based on the MEDLINE search performed by Vo and colleagues [29] who conducted a review aimed to assess the methodological characteristics of mediation analyses conducted in randomized controlled trials between 2017 and 2018. We adapted the search conducted by Vo and colleagues [29] in four ways. First, we searched both the MEDLINE and EMBASE, as EMBASE has been shown to contain many unique references compared to MEDLINE when performing medically-oriented searches [68]. Second, we extended the search period to 5 years, including papers published between January 1st 2015 and December 31st 2019, as estimation methods for causal mediation analysis have been implemented in all major software packages since 2015 [21–28]. Third, in addition to the keywords “mediation analysis”, mediation, and mediator used by Vo and colleagues [29], we also included the following keywords to increase the chances of finding papers that conducted a mediation analysis: “mediation analys*”, mediators, “indirect effect”, “indirect effects”, “causal steps”, “product-of-coefficients”, and “difference-in-coefficients”. Fourth, we searched for observational studies only, as the earlier study performed by Vo and colleagues [29] examined the methodological characteristics of mediation analyses conducted in randomized controlled trials. The MEDLINE (accessed through PubMed) and EMBASE (accessed through embase.com) searches were performed on May 20th 2020. The complete MEDLINE and EMBASE search strategies can be found in supplementary appendix 2.

After removing duplicate records, two authors (JJMR and SJL) independently screened the titles and abstracts of the identified records for eligibility using Rayyan software [69]. Records were eligible for inclusion when published between 2015 and 2019, written in English, based on observational human subjects data, and the title or abstract indicated that it concerned an original research paper in which mediation analysis was performed. Full texts of the eligible records were obtained. When full texts were not available, full texts were requested from the corresponding author by email. Two authors (JJMR and SJL) independently screened the full texts for eligibility. Full texts in which mediation analysis was not performed as one of the primary analysis methods and conference abstracts were excluded, as we expected that these records did not contain a sufficient amount of details on the performed mediation analyses. Disagreements at any stage of the screening process were resolved by a third author (MJV).

A data extraction form was developed and pilot tested by one author, who subsequently extracted data from all eligible papers (JJMR). To ensure the quality of the extracted data, two authors (MJV and SJL) each independently extracted data from a random subsample of 12.5% of the eligible papers, i.e., 25% of the papers in total. Disagreements were resolved through discussion. The data extraction included the mediation analysis method used, publication year, study design, sample size, software used, the number of exposure, mediator, and outcome variables, each variable’s measurement level, use of a path diagram, use of repeated measurements, single or multiple mediator model, the types of estimated regression models, the type of confidence interval for the indirect effect estimates, the reporting of standard errors and *p*-values for the indirect effect estimates, use of effect size measures, inclusion of confounders in the analyses, use of sensitivity analyses for unmeasured confounders, assessment of exposure-mediator interaction, assessment of effect modifiers (i.e., exposure-by-covariate or mediator-by-covariate), and the discussion of the rare outcome assumption for mediation models with a binary or time-to-event outcome estimated based on traditional mediation analysis or regression-based causal mediation analysis. For papers based on longitudinal data we extracted the number of measurement waves included in the analyses and the type of longitudinal mediation model estimated. For multiple mediator models we extracted the type of multiple mediator model and the assessment of mediator-by-mediator interactions. The extracted data were summarized using descriptive statistics stratified by the mediation analysis method used. Categorical variables were summarized using frequencies and percentages, and continuous variables were summarized using medians and interquartile ranges.

## Results

**Fig. 2:**
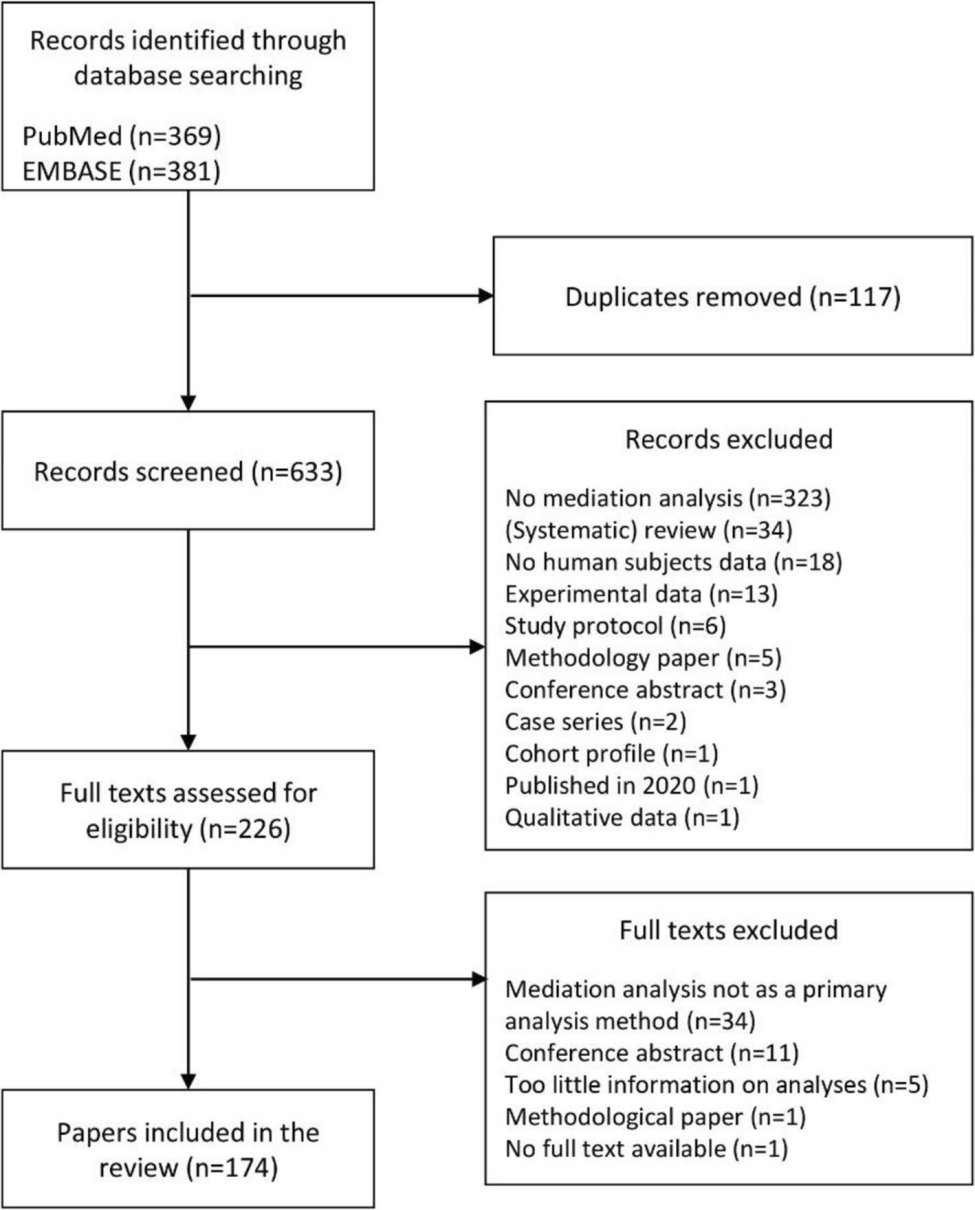
Flow diagram representing the process of identifying papers eligible for the review of the methodological characteristics of mediation analyses performed based on observational epidemiologic studies published between 2015 and 2019

The search returned 369 records through the MEDLINE database and 381 records through the EMBASE database (Fig. 2). After removing duplicates, 633 records remained for the title and abstract screening. Conflicting decisions were made for 25 records (3.9%) and were resolved by a third author. A total of 407 records were excluded after the title and abstract screening, with the most common reason for exclusion being that the title or abstract did not indicate that mediation analysis was performed (*n* = 323). Two hundred twenty-six records were eligible for full-text screening. For one of the eligible records, no full text could be obtained. Conflicting decisions were made for 10 papers (4.4%) and were resolved by a third author. Based on the full text screening, another 43 records were excluded, of which 34 did not perform mediation analysis as one of the primary analyses, 11 were conference abstracts, 5 provided too little information for data extraction, and 1 paper was a methodological study. A total of 174 papers were included in the review. A complete list of included papers can be found in the supplementary appendix 3 and the dataset with the extracted data in supplementary appendix 4.

Table 2 provides an overview of the methodological characteristics of the mediation analyses performed by the studies included in this scoping review. Of the 174 studies included in this scoping review, 123 used traditional mediation analysis (70.7%). Twenty-eight papers (16.1%) used the causal steps method (*n* = 14), the change-in-coefficient method (*n* = 9), or the test of joint significance (*n* = 5). In line with a previous paper, we define the change-in-coefficient method as the assessment of the presence of a mediated effect based on the change in the exposure-outcome coefficient before and after inclusion of the mediator in the model [20]. The test of joint significance is based on the joint statistical significance of the exposure-mediator and mediator-outcome effect estimates. The causal steps method, change-in-coefficient method, and test of joint significance are similar in that they do not provide indirect effect estimates. Therefore, we collapsed the descriptive statistics in Table 2 across these three methods. Twenty-three papers used causal mediation analysis (13.2%), of which 10 used the regression-based estimation approach (43.5%), 7 used the simulation-based estimation approach (30.4%), 4 used natural effects models (17.4%), 1 used numerical integration (4.3%), and for 1 paper it remained unclear which estimation method was used.

**Table 2:** Methodological Characteristics of Mediation Analyses Performed Based on Observational Epidemiologic Studies Published Between 2015 and 2019

Methodological characteristics	Overall (*n* = 174)	Causal steps, change-in-coefficient, and joint significance (*n* = 28)	Traditional mediation analysis (*n* = 123)	Causal mediation analysis (*n* = 23)
Year published, *n* (%)^a^				
2015	21 (12.1)	4 (14.3)	16 (13.0)	1 (4.3)
2016	29 (16.7)	8 (28.6)	16 (13.0)	5 (21.7)
2017	27 (15.5)	2 (7.1)	21 (17.1)	4 (17.4)
2018	47 (27.0)	6 (21.4)	34 (27.6)	7 (30.4)
2019	50 (28.7)	8 (28.6)	36 (29.3)	6 (26.1)
Study design, *n* (%)^a^				
Cross-sectional	84 (48.3)	15 (53.6)	64 (52.0)	5 (21.7)
Case-control	7 (4.0)	1 (3.6)	3 (2.4)	3 (13.0)
Prospective cohort	78 (44.8)	12 (42.9)	53 (43.1)	13 (56.5)
Retrospective cohort	5 (2.9)	0 (0.0)	3 (2.4)	2 (8.7)
Analytical sample size, median (IQR)	428.5 (157.5–2026.0)	443.5 (146.5–1442.3)	326.0 (147.0–935.0)	2249.0 (703.0–9484.0)
Software program used, *n* (%)^a^				
SAS	21 (12.1)	4 (14.3)	9 (7.3)	8 (34.8)
Stata	27 (15.5)	5 (17.9)	18 (14.6)	4 (17.4)
SPSS	67 (38.5)	13 (46.4)	54 (43.9)	0 (0.0)
M*plus*	26 (14.9)	1 (3.6)	25 (20.3)	0 (0.0)
R	14 (8.0)	0 (0.0)	5 (4.1)	9 (39.1)
LISREL	1 (0.6)	0 (0.0)	1 (0.8)	0 (0.0)
Unclear, multiple programs used	5 (2.9)	1 (3.6)	4 (3.3)	0 (0.0)
Not mentioned	13 (7.5)	4 (14.3)	7 (5.7)	2 (8.7)
Number of exposures, *n* (%)^a^				
1	116 (66.7)	19 (67.9)	81 (65.9)	16 (69.6)
2	35 (20.1)	3 (10.7)	26 (21.1)	6 (26.1)
3 or more (max. 14)	23 (13.2)	6 (21.4)	16 (13.0)	1 (4.3)
Type of exposure, *n* (% of studies)^b^				
Continuous	105 (60.3)	15 (53.6)	84 (68.3)	6 (26.1)
Binary	51 (29.3)	8 (28.6)	28 (22.8)	15 (65.2)
Categorical	20 (11.5)	5 (17.9)	11 (8.9)	4 (17.4)
Latent	10 (5.7)	2 (7.1)	8 (34.8)	0 (0.0)
Number of mediators, *n* (%)^a^				
1	86 (49.4)	14 (50.0)	59 (48.0)	13 (56.5)
2	35 (20.1)	5 (17.9)	28 (22.8)	2 (8.7)
3 or more (max. 19)	53 (30.5)	9 (32.1)	36 (29.3)	8 (34.8)
Type of mediator, *n* (% of studies)^b^				
Continuous	136 (78.2)	19 (67.9)	103 (83.7)	14 (60.9)
Binary	31 (17.8)	10 (35.7)	13 (10.6)	8 (34.8)
Categorical	9 (5.2)	3 (10.7)	3 (2.4)	3 (13.0)
Count	1 (0.6)	0 (0.0)	0 (0.0)	1 (4.3)
Time-to-event	2 (1.1)	0 (0.0)	2 (1.6)	0 (0.0)
Latent	10 (5.7)	3 (10.7)	7 (5.7)	0 (0.0)
Number of outcomes, *n* (%)^a^				
1	126 (72.4)	22 (78.6)	88 (71.5)	16 (69.6)
2	26 (15.0)	2 (7.1)	22 (17.9)	2 (8.7)
3 or more (max. 8)	22 (12.6)	4 (14.3)	13 (10.6)	5 (21.6)
Type of outcome, *n* (% of studies)^b^				
Continuous	110 (63.2)	18 (64.3)	86 (69.9)	6 (26.1)
Binary	47 (27.0)	6 (21.4)	27 (22.0)	14 (60.9)
Categorical	6 (3.4)	2 (7.1)	3 (2.4)	1 (4.3)
Count	4 (2.3)	0 (0.0)	2 (1.6)	2 (8.7)
Time-to-event	8 (4.6)	3 (10.7)	3 (2.4)	2 (8.7)
Latent	10 (5.7)	3 (10.7)	7 (5.7)	0 (0.0)
Diagram of model included, *n* (%)^a^				
Yes	130 (74.7)	14 (50.0)	106 (86.2)	10 (43.5)
No	44 (25.3)	14 (50.0)	17 (13.8)	13 (56.5)
Repeated measurements, *n* (%)^a^				
Yes	41 (23.6)	6 (21.4)	28 (22.8)	7 (30.4)
No	132 (75.9)	22 (78.6)	94 (76.4)	16 (69.6)
Unclear	1 (0.6)	0 (0.0)	1 (0.8)	0 (0.0)
Type of mediation model, *n* (%)^a^				
Single mediator	114 (65.5)	20 (71.4)	74 (60.2)	20 (87.0)
Multiple mediator	41 (23.6)	5 (17.9)	34 (27.6)	2 (8.7)
Both single and multiple mediator	16 (9.2)	3 (10.7)	12 (9.8)	1 (4.3)
Unclear	3 (1.7)	0 (0.0)	3 (2.4)	0 (0.0)
Regression for the mediator equation, *n* (% of studies)^b^				
Linear	122 (70.1)	14 (50.0)	98 (79.7)	10 (43.4)
Logistic	15 (8.6)	2 (7.1)	7 (5.7)	6 (26.1)
Poisson	1 (0.6)	0 (0.0)	0 (0.0)	1 (4.3)
Cox proportional hazards	2 (1.1)	0 (0.0)	2 (1.6)	0 (0.0)
Multilevel linear	7 (4.0)	0 (0.0)	7 (5.7)	0 (0.0)
Multilevel logistic	1 (0.6)	1 (3.6)	0 (0.0)	0 (0.0)
Unclear	16 (9.2)	2 (7.1)	8 (6.5)	6 (26.1)
Not estimated	12 (6.9)	9 (32.1)	3 (2.4)	0 (0.0)
Regression for the outcome equation, *n* (% of studies)^b^				
Linear	109 (62.6)	17 (60.7)	87 (70.7)	5 (21.7)
Logistic	39 (22.4)	5 (17.9)	23 (18.7)	11 (47.8)
Probit	1 (0.6)	0 (0.0)	1 (0.8)	0 (0.0)
Log-linear	1 (0.6)	0 (0.0)	0 (0.0)	1 (4.3)
Multinomial logistic	1 (0.6)	1 (3.6)	2 (1.6)	0 (0.0)
Poisson	3 (1.7)	0 (0.0)	0 (0.0)	3 (13.0)
Negative binomial	3 (1.7)	0 (0.0)	1 (0.8)	2 (8.7)
Cox proportional hazards	10 (5.7)	4 (14.3)	4 (3.3)	1 (4.3)
Additive hazards model	1 (0.6)	0 (0.0)	0 (0.0)	1 (4.3)
Multilevel linear	9 (5.2)	2 (7.1)	7 (5.7)	0 (0.0)
Multilevel logistic	1 (0.6)	0 (0.0)	1 (0.8)	0 (0.0)
Multilevel log-linear	1 (0.6)	0 (0.0)	1 (0.8)	0 (0.0)
Unclear	3 (1.7)	1 (3.6)	1 (0.8)	1 (4.3)
Type of confidence interval for the indirect effect, *n* (%)^a^				
Normal-based	7 (4.0)	0 (0.0)	6 (4.9)	1 (4.3)
Percentile bootstrap	7 (4.0)	0 (0.0)	5 (4.1)	2 (8.7)
Bias-corrected bootstrap	35 (20.1)	0 (0.0)	35 (28.5)	0 (0.0)
Bias-corrected and accelerated bootstrap	2 (1.1)	0 (0.0)	2 (1.6)	0 (0.0)
Percentile and bias-corrected and accelerated bootstrap	1 (0.6)	0 (0.0)	1 (0.8)	0 (0.0)
Bootstrap, not specified	36 (20.7)	1 (3.6)	28 (22.8)	7 (30.4)
Distribution of the product	3 (1.7)	0 (0.0)	3 (2.4)	0 (0.0)
Monte Carlo	2 (1.1)	0 (0.0)	2 (1.6)	0 (0.0)
Bayesian credible intervals	3 (1.7)	0 (0.0)	3 (2.4)	0 (0.0)
Unclear	18 (10.3)	0 (0.0)	8 (6.5)	10 (43.5)
Not reported	60 (34.5)	27 (96.4)	30 (24.4)	3 (13.0)
Standard error for indirect effect, *n* (%)^a^				
Yes	37 (21.3)	0 (0.0)	35 (28.5)	2 (8.7)
No	137 (78.7)	28 (100.0)	88 (71.5)	21 (91.3)
Statistical test for indirect effect, *n* (%)^a^				
Yes	62 (35.6)	7 (25.0)	46 (37.4)	9 (39.1)
No	112 (64.4)	21 (75.0)	77 (62.6)	14 (58.3)
Effect size measure, *n* (%)^a^				
Proportion mediated	65 (37.4)	3 (10.7)	43 (35.0)	19 (82.6)
Standardized effect	6 (3.4)	0 (0.0)	6 (4.9)	0 (0.0)
No effect size measure	103 (59.2)	25 (89.3)	74 (60.2)	4 (17.4)
Inclusion of confounders, *n* (%)^a^				
Yes	125 (71.8)	19 (67.9)	88 (71.5)	18 (78.3)
No	41 (23.6)	8 (28.6)	31 (25.2)	2 (8.7)
Unclear	8 (4.6)	1 (3.6)	4 (3.3)	3 (13.0)
Sensitivity analyses for unmeasured confounders, *n* (%)^a^				
Yes	3 (1.7)	0 (0.0)	0 (0.0)	3 (13.0)
No	170 (97.7)	28 (100.0)	123 (0.0)	19 (82.6)
No, but discussed as unnecessary	1 (0.6)	0 (0.0)	0 (0.0)	1 (4.3)
Effect modifiers considered, *n* (%)^a^				
Yes, a priori stratification	10 (5.7)	3 (10.7)	5 (4.1)	2 (8.7)
Yes, through an interaction term	28 (16.1)	6 (21.4)	19 (15.4)	3 (13.0)
No	136 (78.2)	19 (67.9)	99 (80.5)	18 (78.3)
Assessment of exposure-mediator interaction, *n* (%)^a^				
Yes	17 (9.8)	3 (10.7)	5 (4.1)	9 (39.1)
No	157 (90.2)	25 (89.3)	118 (95.9)	14 (60.9)

^a^ The percentages are computed based on the column frequencies

^b^ Because some studies considered multiple exposure, mediator, and outcome variables, the total number is higher than the number of studies. The percentages reflect the percentages of the total number of studies, e.g., 60.3% of the studies included analyses with a continuous exposure variable versus 39.7% which did not include analyses with a continuous exposure variable

Twenty-one studies were published in 2015 (12.1%), 29 in 2016 (16.7%), 27 in 2017 (15.5%), 47 in 2018 (27.0%), and 50 in 2019 (28.7%). The cross-sectional study design was the most common (48.3%), followed by the prospective cohort design (44.8%). The case-control design and retrospective cohort design were less common (4.0 and 2.9% respectively). Studies using causal mediation analysis were more often based on a case-control design and less often on a cross-sectional design than studies using other mediation analysis methods. The median number of participants eligible for analyses was 428.5 (interquartile range: 157.5–2026.0). SPSS was most commonly used to perform mediation analysis (38.5%), followed by Stata (15.5%), M*plus* (14.9%), SAS (12.1%), R (8.0%), and LISREL (0.6%). Thirteen studies did not mention the used software program (7.5%). Five studies mentioned the use of multiple software programs (2.9%).

Most studies considered one exposure variable (66.7%) or one outcome variable (72.4%). Eighty-six studies considered one mediator variable (49.4%), 35 studies considered two mediator variables (20.1%), and 53 studies considered three or more mediator variables (30.5%). The majority of studies performed mediation analysis based on continuous exposure, mediator, and outcome variables. Causal mediation analysis was used relatively often to analyze binary outcomes, but was never used to analyze latent variables. One-hundred-thirty studies reported a diagram of the mediation model (74.7%). Ten of these studies included confounders in the diagram (7.7%).

Forty-one studies performed mediation analysis based on repeated measurements of the variables in the mediation model (23.6%). The median amount of measurement waves among these studies was 2.0 (IQR: 2.0–4.0). The methodology used to analyze repeated measurements varied from adjustment for first-wave measurements to more complicated models, such as cross-lagged panel models, latent growth curve models, and multilevel models. A detailed table of the used methods to estimate mediation models based on repeated measurements can be found in supplementary appendix 5.

One-hundred-fourteen studies reported single mediator models only (65.5%), 41 studies reported multiple mediator models only (23.6%), and 16 studies reported both single and multiple mediator models (9.2%). Of the 16 studies reporting both single and multiple mediator models, 10 studies reported parallel multiple mediator models in addition to single mediator models (62.5%), 5 studies reported serial multiple mediator models in addition to single mediator models (31.3%), and 1 study reported both parallel and serial multiple mediator models in additional to single mediator models (6.3%). Of all 57 studies reporting multiple mediator models, 37 studies reported parallel multiple mediator models (64.9%), 18 studies reported serial multiple mediator models (31.6%), and 2 studies reported both parallel and serial multiple mediator models (3.5%). None of these studies reported that they assessed mediator-by-mediator interactions. Most studies using causal mediation analysis reported single mediator models (87.5%).

Most studies used linear regression to estimate the mediator and outcome eqs. (70.1 and 62.6%, respectively). Of the 47 studies using a (traditional or causal) regression-based estimation approach for models with a binary or time-to-event outcome, 1 study discussed the rare outcome assumption (2.1%) and 3 studies estimated effects on the relative-risk scale or risk-difference scale (6.4%). The latter 4 studies all used causal mediation analysis. Of the 123 studies using traditional mediation analysis, 98 used the product-of-coefficients estimator (79.7%), 3 used the difference-in-coefficients estimator (2.4%), 16 did not specify the used method for calculating the indirect effect (13.0%), and 6 did not report indirect effect estimates (4.9%). Bias-corrected bootstrap confidence intervals were the most commonly reported type of confidence interval for the indirect effect estimates (20.1%). Thirty-seven studies reported a standard error for the indirect effect estimate (21.3%) and 62 studies reported a *p*-value for the indirect effect estimate (35.6%). The proportion mediated was the most commonly used effect size measure (37.5%). Six studies determined effect sizes by comparing standardized effect estimates with Cohen’s *d* (3.4%).

Most studies included confounders in the mediation analyses (71.8%). Only 3 studies performed sensitivity analyses for unmeasured confounders (1.7%), and 1 study discussed the no-unmeasured confounder assumptions and concluded that the estimated models were adjusted for all important confounders (0.6%). All studies performing or discussing sensitivity analyses for unmeasured confounders used causal mediation analysis. Most studies did not investigate moderated mediation (78.2%). Ten studies stratified the analyses a priori based on an effect modifier (5.7%). Twenty-eight studies investigated moderation by including interaction terms in the models (16.1%), of which 17 studies reported that the coefficient for the interaction term was not statistically significant. Of the 11 studies with statistically significant interaction effects, 5 studies reported overall effects (45.5%), 3 studies reported the estimated coefficient for the interaction term (27.3%), and 3 studies stratified the analyses based on the effect modifier (27.3%). Of the 17 studies that tested exposure-mediator interaction, 8 reported a statistically significant interaction (35.3%). Only 2 of these studies incorporated the exposure-mediator interaction in the effect estimates. Both of these studies used causal mediation analysis to estimate the effects.

## Discussion

The aim of this paper was to review the methodological characteristics of mediation analyses performed in observational epidemiologic studies published between 2015 and 2019 and to provide recommendations for the application of mediation analyses in future studies. This scoping review showed that traditional mediation analysis was frequently used in observational studies published between 2015 and 2019. A minority of studies used causal mediation analysis and compared to the other mediation analysis methods, causal mediation analysis was less often used to analyze relatively complex mediation models, such as models with latent variables and multiple mediator models. The majority of studies included measured confounders in their mediation analyses. However, sensitivity analyses for unmeasured confounding, exposure-mediator interaction, and the rare outcome assumption for binary and time-to-event outcomes were only discussed in a few papers, most of which used causal mediation analysis. Based on the findings in this scoping review, the next section provides recommendations for conducting mediation analysis based on real-life data.

### Recommendations for conducting mediation analysis

#### Mediation analysis method

Although the causal steps method, change-in-coefficient method, and the test of joint significance are relatively old methods for mediation analysis, they were still applied in over 15 % of the papers included in this scoping review. These methods are not preferred for mediation analysis, as they do not necessarily provide mediated effect estimates [70]. Furthermore, the causal steps method and the test of joint significance rely completely on the statistical significance of the estimated coefficients. The causal steps method does therefore not account for inconsistent mediation models in which the direct and indirect effect estimates have opposite signs, where the total effect estimate can approach zero [11, 34, 71]. Therefore, mediation effects might be missed when relying on the causal steps criteria. The change-in-coefficient method may result in biased conclusions for models with a binary or time-to-event outcome as the change in the coefficient may reflect a change in the scales of the effect estimates (i.e., non-collapsibility) instead of mediation [41, 44, 72].

Although traditional and causal mediation analysis provide the same effect estimates for some models, causal mediation analysis is generally preferred over traditional mediation analysis. Causal mediation analysis explicitly lays out all assumptions needed for the causal interpretation of the effect estimates [19, 73]. Although some of these causal assumptions are the same as the parametric assumptions posed by the other mediation analysis methods, causal mediation analysis also provides guidance for when these assumptions do not hold [74]. For example, when there are unmeasured confounders, sensitivity analyses might be used to assess how the effect estimates change based on a range of plausible assumptions regarding the magnitude of the effect of the confounder on the variables in the mediation model [53, 75–77]. The clarification of the causal assumptions is an important contribution of causal mediation analysis, as mediation models are inherently causal models.

Causal mediation analysis is also preferred over traditional mediation analysis as it provides causal effect definitions that can be used to estimate causal effects for any mediation model [45]. In contrast, the traditional estimators were originally derived based on linear regression coefficients [9], and are also applied based on the coefficients from other types of regression models, such as logistic regression and Cox regression [12, 78]. Provided that the no (unmeasured) confounding assumptions hold, traditional mediation analysis provides causal effect estimates for mediation models estimated with linear regression [16, 17, 19]. However, when eq. 1 is estimated with linear regression and eq. 2 is estimated with non-linear regression, e.g., logistic regression or Cox proportional hazards regression, traditional and causal mediation analysis only provide the same effect estimates when the mediator follows a normal distribution, the outcome is rare, and interactions are absent [17, 19, 79]. When there is exposure-mediator interaction in a mediation model with a binary outcome variable, the traditional direct effect estimates map onto the causal CDE estimates, rather than the causal PNDE and TNDE estimates [18].

#### Parametric and causal assumptions

It is generally recommended to assess and discuss the relevant parametric and causal assumptions. The no (unmeasured) confounding assumptions are essential to ensure a causal interpretation of the effect estimates and are especially relevant for observational studies, as all paths in the mediation model are observational and adjustment for confounders is essential to ensure the causal interpretation of the effect estimates. Directed acyclic graphs (DAGs) can be used to help determine the confounders of the paths in the mediation model, as DAGs visualize the causal paths in the mediation model, including the confounders of these paths [49, 80]. The majority of studies in this review reported a path diagram of the mediation model, but these path diagrams are different from DAGs, as path diagrams typically represent the statistical model, while DAGs represent the theoretical model including (unmeasured) confounders of each pathway in the mediation model [81]. Future studies could clarify the causal structure of their mediation model by reporting a DAG, possibly in addition to the path diagram. The potential impact of unmeasured confounders on the effect estimates can be assessed through sensitivity analyses [53, 77]. When the fourth no confounding assumption is violated, multiple mediator models can be estimated to take into account the additional mediator variables [25, 61–63].

The presence of covariate-exposure, covariate-mediator, exposure-mediator and mediator-mediator interactions can be assessed by adding interaction terms to the statistical models. This is important because the overall effects ignore important information on the direct and indirect effect estimates when statistically significant or clinically relevant interactions are not taken into account [28, 82].

Finally, it is important to assess the rare-outcome assumption when using a regression-based estimation approach for the analysis of a mediation model with a binary or time-to-event outcome, as the effect estimates on the odds-ratio scale and hazard-ratio scale only have a causal interpretation when the outcome prevalence is low across all strata of the exposure and mediator variables [83]. When the rare-outcome assumption is violated it is advised to estimate the effects for models with a binary outcome with log-linear regression and the effects for models with a time-to-event outcome with accelerated failure time models [28, 57].

#### Statistical inference

Over one-third of the papers in this scoping review determined the statistical significance of the indirect effect estimate based on a *z*-test, which has relatively low power to detect a statistically significant indirect effect [35, 36]. Instead, it is recommended to determine the statistical significance of the indirect effect estimate based on a confidence interval that takes into account the nonnormal sampling distribution of the indirect effect estimator, such as the distribution of the product confidence interval, Monte Carlo confidence interval, and bootstrap confidence intervals, as these have higher power to detect a statistically significant indirect effect [34, 36, 38, 84–86]. Although the bias-corrected bootstrap confidence interval was the most often reported confidence interval in the studies in this scoping review, percentile bootstrap confidence intervals generally perform best in terms of the balance between type I and type II error rates [36, 87, 88].

#### Relative effect size measures

In addition to the (natural) indirect effect estimates, over one-third of the studies in this scoping review reported the proportion mediated as a relative effect size measure for the mediated effect. Although the proportion mediated has an intuitive interpretation, it does suffer from a few important limitations. First, a previous simulation study showed that the proportion mediated is unstable in samples of less than 500 participants [13]. In this review, 21 papers with a sample of less than 500 participants estimated the proportion mediated. Second, the estimate of the proportion mediated can be below zero or above one when the mediation model is inconsistent [2, 3]. In this situation, the proportion mediated does not have a meaningful interpretation. Third, the estimate of the proportion mediated can be misleading when the underlying effect estimates are small and clinically irrelevant, as the estimate of the proportion mediated can still be large in this situation. Therefore, it is advised to only estimate the proportion mediated when none of the aforementioned situations apply. If the aforementioned situations do apply, it may suffice to only report the natural indirect effect estimate with a confidence interval. However, when the indirect effect is estimated based on variables without a naturally meaningful interpretation, such as variables measured on a Likert scale, researchers may alternatively determine the relative effect size by comparing the standardized indirect effect estimate to Cohen’s *d* [89, 90].

### Recommendations for enhancing the uptake of causal mediation analysis

Although most of the seminal articles on causal mediation analysis were published between 2009 and 2012 [45, 46, 53, 56], and various causal mediation software packages have been developed in the last decade [21–26, 28], the uptake of causal mediation analysis in applied research remains relatively low. A first reason for this low uptake might be the high level of technical details in the causal mediation analysis literature [20, 29]. To enhance the uptake of causal mediation analysis, Vo et al. [29] suggested that there is a need for detailed tutorial papers. As binary and time-to-event outcomes are common in epidemiology and causal mediation analysis clarifies the ambiguities that arise when these outcomes are analyzed with traditional mediation analysis, future tutorial papers could demonstrate the application of causal estimators and the interpretation of causal effect estimates based on real-life data for models with non-continuous mediator variables or non-continuous outcome variables. Another potential topic for a tutorial paper could be the demonstration of the importance of testing the plausibility of the causal assumptions, as this review and previous reviews found that most studies fail to address the plausibility of all causal assumptions [20, 29, 91].

A second reason for the low uptake of causal mediation analysis might be that currently available causal mediation software packages facilitate the estimation of causal effects for a limited range of mediation models. The uptake of causal mediation analysis can also be enhanced through the expansion of current software packages and/or the development of new software packages that facilitate causal effect estimation for a wider range of more complicated mediation models, such as models with latent variables and multiple mediator models. To date, only M*plus* facilitates the estimation of causal effects for mediation models with latent variables and the causal effect estimation for multiple mediator models is only supported by the Mediation and Medflex packages in R [23, 25–27]. Also, the causal effect estimation for multilevel and longitudinal mediation models is limitedly supported by the currently available software packages and warrants attention in future software development [27].

### Strengths and limitations

This scoping review assessed the methodological characteristics of mediation analyses published based on observational data. Observational data is common in the field of epidemiology and mediation analysis is becoming an increasingly popular method to analyze observational data. Two previously published reviews also reported that traditional mediation analysis is the most frequently used mediation analysis method, but one of these reviews focused on the analysis of experimental data [29], and the other on mediation models with time-to-event outcomes [20]. This scoping review was not restricted to specific types of mediation models, providing insight in the use of mediation analysis methods across a range of model characteristics. Another strength of this review is that it covered a relatively wide range of publication years to gain insight into the uptake of causal mediation analysis in recent years. Based on the current practices observed in this scoping review, we provided recommendations for applied researchers who wish to apply mediation analysis to their data.

A limitation of this study is that the results might not be generalizable to all observational mediation analyses published between 2015 and 2019, as we only searched two databases and the search strategy was limited to the title, abstract and keywords of the papers. Therefore, it is likely that not all observational mediation analyses published between 2015 and 2019 were identified by our search. However, the goal of our paper was to provide insight into the methodological characteristics of mediation analysis methods used to analyze observational data. Even though this scoping review may not have included *all* observational mediation analyses published between 2015 and 2019, the results demonstrate large heterogeneity in the mediation analysis methods used to analyze observational data. Based on the findings in this scoping review, we were able to provide recommendations to improve the quality of future mediation analyses. Furthermore, compared to the previously published review by Vo and colleagues [29] who reviewed the methodological characteristics of mediation analysis methods applied in randomized controlled trials, we used a more extensive search term, a longer search period, and we searched both the MEDLINE and EMBASE databases. MEDLINE and EMBASE are two of the largest databases for epidemiological publications and with 174 included papers this is one of the largest reviews on mediation analysis methods so far [20, 29, 91, 92].

Another limitation is that the studies included in this scoping review might not have been able to report all aspects of their mediation analyses due to journal requirements such as word limits. For example, although the no (unmeasured) confounding assumptions are of critical importance in mediation analysis, the studies in this review generally provided little information on the causal theory underlying the confounder selection. That is, information was generally lacking on the specific pathways that might be confounded by each of the confounders. Journal requirements might therefore partially explain the large heterogeneity in the reporting of mediation analyses observed in this scoping review and in previous reviews [20, 29, 91, 93]. The transparency in the reporting of future mediation analyses will likely be enhanced by the guideline for the reporting of mediation analyses that was recently published [94].

## Conclusion

Mediation analysis is becoming increasingly popular in the field of epidemiology, as it can be used to gain insight into mechanisms of disease development. Even though causal mediation analysis is the generally preferred method for mediation analysis, we showed that traditional mediation analysis is still frequently applied in practice. We recommend that researchers use causal mediation analysis and assess the plausibility of relevant causal assumptions to ensure the causal interpretation of the direct and indirect effect estimates. Furthermore, the uptake of causal mediation analysis could be enhanced through tutorial papers and the development of software packages that facilitate the estimation of causal effects for relatively complicated mediation models.

## Supplementary Information

**Additional file 1: Supplementary appendix 1.** PRISMA-ScR checklist.

**Additional file 2: Supplementary appendix 2.** The PubMed and EMBASE search strategies.

**Additional file 3: Supplementary appendix 3.** List of papers included in the scoping review.

**Additional file 4: Supplementary appendix 4.** Dataset with extracted data.

**Additional file 5: Supplementary appendix 5.** Overview of mediation analysis methods used to analyze repeated measurements in the included papers.

## Data Availability

The dataset supporting the conclusions of this article is included within the article and its additional files.

## Abbreviations

BMI: body mass index
CDE: controlled direct effect
DAG: directed acyclic graph
PNIE: pure natural indirect effect
PNDE: pure natural direct effect
PRISMA: preferred reporting items for systematic reviews and meta-analyses
TE: total effect
TNIE: total natural indirect effect
TNDE: total natural direct effect
