# Supplementary material for: Mediation analysis methods used in observational research: a scoping review and recommendations
Source: BMC Med Res Methodol. 2021 Oct 25;21:226. doi: 10.1186/s12874-021-01426-3 (PMC8543973; doi:10.1186/s12874-021-01426-3)
Supplement: Supplementary file 2 — Additional file 2: Supplementary appendix 2. The PubMed and EMBASE search strategies. [file 12874_2021_1426_MOESM2_ESM.docx]

**Supplementary appendix 2: The PubMed and EMBASE search strategies**

**Pubmed search strategy**

(“Mediation analys*”[tiab] OR Mediation[tiab] OR Mediator[tiab] OR Mediators[tiab] OR “Indirect effect”[tiab] OR “Indirect effects”[tiab] OR “Causal steps”[tiab] OR “Product-of-coefficients”[tiab] OR “Difference-in-coefficients”[tiab]) AND (“observational study”[Publication Type]) AND ("2015/01/01"[Date - Publication] : "2019/12/31"[Date - Publication])

**EMBASE search strategy**

(('mediation analysis'/exp OR 'mediation analyses':ti,ab,kw OR 'mediation'/exp OR 'mediator'/exp OR 'mediators':ti,ab,kw OR 'indirect effect':ti,ab,kw OR 'indirect effects':ti,ab,kw OR 'causal steps':ti,ab,kw OR 'product-of-coefficients':ti,ab,kw OR 'difference-in-coefficients':ti,ab,kw) AND [embase]/lim) AND ('observational study'/exp AND [embase]/lim) AND ([2015-2019]/py)
