# Supplementary material for: Mediation analysis methods used in observational research: a scoping review and recommendations
Source: BMC Med Res Methodol. 2021 Oct 25;21:226. doi: 10.1186/s12874-021-01426-3 (PMC8543973; doi:10.1186/s12874-021-01426-3)
Supplement: Supplementary file 3 — Additional file 3: Supplementary appendix 3. List of papers included in the scoping review. [file 12874_2021_1426_MOESM3_ESM.docx]

**Supplementary appendix 3: List of papers included in the scoping review** (1-174)

1. Abdala GA, Kimura M, Duarte YA, Lebrão ML, dos Santos B. Religiousness and health-related quality of life of older adults. Rev Saude Publica. 2015;49:55.

2. Freitas TH, Andreoulakis E, Alves GS, Miranda HL, Braga LL, Hyphantis T, et al. Associations of sense of coherence with psychological distress and quality of life in inflammatory bowel disease. World J Gastroenterol. 2015;21(21):6713-27.

3. Friedman MR, Stall R, Silvestre AJ, Wei C, Shoptaw S, Herrick A, et al. Effects of syndemics on HIV viral load and medication adherence in the multicentre AIDS cohort study. Aids. 2015;29(9):1087-96.

4. Gau SS, Tseng WL, Tseng WY, Wu YH, Lo YC. Association between microstructural integrity of frontostriatal tracts and school functioning: ADHD symptoms and executive function as mediators. Psychol Med. 2015;45(3):529-43.

5. Gray WN, Boyle SL, Graef DM, Janicke DM, Jolley CD, Denson LA, et al. Health-related quality of life in youth with Crohn disease: Role of disease activity and parenting stress. J Pediatr Gastr Nutr. 2015;60(6):749-53.

6. Hildebrand M, Kolle E, Hansen BH, Collings PJ, Wijndaele K, Kordas K, et al. Association between birth weight and objectively measured sedentary time is mediated by central adiposity: Data in 10,793 youth from the International Children's Accelerometry Database. Am J of Clin Nutr. 2015;101(5):983-90.

7. Kim S, Noh D, Park SI. Mediating effect of stress on the association between early trauma and psychological distress in Korean college students: a cross-sectional observational study. J Psychiatr Ment Health Nurs. 2015;22(10):784-91.

8. Ling J, Robbins LB, McCarthy VL, Speck BJ. Psychosocial determinants of physical activity in children attending afterschool programs: a path analysis. Nurs Res. 2015;64(3):190-9.

9. Lord JH, Young MT, Gruhn MA, Grey M, Delamater AM, Jaser SS. Effect of race and marital status on mothers' observed parenting and adolescent adjustment in youth with type 1 diabetes. J Pediatr Psychol. 2015;40(1):132-43.

10. Müller R, Peter C, Cieza A, Post MW, Van Leeuwen CM, Werner CS, et al. Social skills: A resource for more social support, lower depression levels, higher quality of life, and participation in individuals with spinal cord injury? Arch Phys Med Rehab. 2015;96(3):447-55.

11. O'Reilly K, Donohoe G, Coyle C, O'Sullivan D, Rowe A, Losty M, et al. Prospective cohort study of the relationship between neuro-cognition, social cognition and violence in forensic patients with schizophrenia and schizoaffective disorder. BMC Psychiatry. 2015;15:155.

12. Panattoni L, Stone A, Chung S, Tai-Seale M. Patients report better satisfaction with part-time primary care physicians, despite less continuity of care and access. J Gen Intern Med. 2015;30(3):327-33.

13. Petre B, Torbey S, Griffith JW, De Oliveira G, Herrmann K, Mansour A, et al. Smoking increases risk of pain chronification through shared corticostriatal circuitry. Hum Brain Mapp. 2015;36(2):683-94.

14. Romero-Maroto M, Santos-Puerta N, González Olmo MJ, Peñacoba-Puente C. The impact of dental appearance and anxiety on self-esteem in adult orthodontic patients. Orthod Craniofac Res. 2015;18(3):143-55.

15. Song Y, Huang YT, Song Y, Hevener AL, Ryckman KK, Qi L, et al. Birthweight, mediating biomarkers and the development of type 2 diabetes later in life: a prospective study of multi-ethnic women. Diabetologia. 2015.

16. Taylor BD, Ness RB, Olsen J, Hougaard DM, Skogstrand K, Roberts JM, et al. Serum leptin measured in early pregnancy is higher in women with preeclampsia compared with normotensive pregnant women. Hypertension. 2015;65(3):594-9.

17. Umeda M, Corbin LW, Maluf KS. Pain mediates the association between physical activity and the impact of fibromyalgia on daily function. Clin Rheumatol. 2015;34(1):143-9.

18. van Nies JA, van Steenbergen HW, Krabben A, Stomp W, Huizinga TW, Reijnierse M, et al. Evaluating processes underlying the predictive value of baseline erosions for future radiological damage in early rheumatoid arthritis. Ann Rheum Dis. 2015;74(5):883-9.

19. Varbo A, Benn M, Smith GD, Timpson NJ, Tybjaerg-Hansen A, Nordestgaard BG. Remnant cholesterol, low-density lipoprotein cholesterol, and blood pressure as mediators from obesity to ischemic heart disease. Circ Res. 2015;116(4):665-73.

20. Xu H, Sjögren P, Ärnlöv J, Banerjee T, Cederholm T, Risérus U, et al. A proinflammatory diet is associated with systemic inflammation and reduced kidney function in elderly adults. J Nutr. 2015;145(4):729-35.

21. Xu T, Brandmaier S, Messias AC, Herder C, Draisma HH, Demirkan A, et al. Effects of metformin on metabolite profiles and LDL cholesterol in patients with type 2 diabetes. Diabetes Care. 2015;38(10):1858-67.

22. Barker DH, Quittner AL. Parental Depression and Pancreatic Enzymes Adherence in Children With Cystic Fibrosis. Pediatrics. 2016;137(2):e20152296.

23. Chin WY, Choi EP, Wan EY, Lam CL. Health-related quality of life mediates associations between multi-morbidity and depressive symptoms in Chinese primary care patients. Fam Pract. 2016;33(1):61-8.

24. Choi EP, Lam CL, Chin WY. Mental Health Mediating the Relationship Between Symptom Severity and Health-Related Quality of Life in Patients with Lower Urinary Tract Symptoms. Low Urin Tract Symptoms. 2016;8(3):141-9.

25. Christian LM, Blair LM, Porter K, Lower M, Cole RM, Belury MA. Polyunsaturated fatty acid (PUFA) status in pregnant women: Associations with sleep quality, inflammation, and length of gestation. PLoS ONE. 2016;11(2).

26. Cooper LL, Woodard T, Sigurdsson S, Van Buchem MA, Torjesen AA, Inker LA, et al. Cerebrovascular Damage Mediates Relations Between Aortic Stiffness and Memory. Hypertension. 2016;67(1):176-82.

27. Hartman L, van Dongen JM, Hildebrandt VH, Strijk JE. The Role of Vitality in the Relationship Between a Healthy Lifestyle and Societal Costs of Health Care and Lost Productivity: A Mediation Analysis. Am J Health Promot. 2016;30(6):465-74.

28. Hendrikx J, Kievit W, Fransen J, van Riel PL. The influence of patient perceptions of disease on medication intensification in daily practice. Rheumatology 2016;55(11):1938-45.

29. Howell BA, Long JB, Edelman EJ, McGinnis KA, Rimland D, Fiellin DA, et al. Incarceration History and Uncontrolled Blood Pressure in a Multi-Site Cohort. J Gen Intern Med. 2016;31(12):1496-502.

30. Kamstrup PR, Nordestgaard BG. Elevated lipoprotein(a) levels, LPA risk genotypes, and increased risk of heart failure in the general population. JACC: Heart Failure. 2016;4(1):78-87.

31. Klimentidis YC, Arora A, Chougule A, Zhou J, Raichlen DA. FTO association and interaction with time spent sitting. Int J Obes (Lond). 2016;40(3):411-6.

32. Kouimtsidis C, Stahl D, West R, Drummond C. Path analysis of cognitive behavioral models in smoking: what is the relationship between concepts involved? J Subst Use. 2016;21(5):553-7.

33. Laslett LL, Otahal P, Hensor EM, Kingsbury SR, Conaghan PG. Knee Pain Predicts Subsequent Shoulder Pain and the Association Is Mediated by Leg Weakness: Longitudinal Observational Data from the Osteoarthritis Initiative. J Rheumatol. 2016;43(11):2049-55.

34. Lau EY, Saunders RP, Pate RR. Factors Influencing Implementation of a Physical Activity Intervention in Residential Children's Homes. Prev Sci. 2016;17(8):1002-11.

35. Marti A, Boes S, Lay V, Escorpizo R, Trezzini B. The association between chronological age, age at injury and employment: Is there a mediating effect of secondary health conditions? Spinal Cord. 2016;54(3):239-44.

36. Matthews MA, Aschner JL, Stark AR, Moore PE, Slaughter JC, Steele S, et al. Increasing F2-isoprostanes in the first month after birth predicts poor respiratory and neurodevelopmental outcomes in very preterm infants. J Perinatol. 2016;36(9):779-83.

37. Mommersteeg PM, Widdershoven JW, Aarnoudse W, Denollet J. Personality subtypes and chest pain in patients with nonobstructive coronary artery disease from the TweeSteden Mild Stenosis study: mediating effect of anxiety and depression. Eur J Pain. 2016;20(3):427-37.

38. Montero D, Houben AJ, Koster A, Muris DM, Schram MT, Gronenschild EH, et al. Physical Activity Is Associated With Glucose Tolerance Independent of Microvascular Function: The Maastricht Study. J Clin Endocrinol Metab. 2016;101(9):3324-32.

39. Nabe-Nielsen K, Grynderup MB, Lange T, Andersen JH, Bonde JP, Conway PM, et al. The role of poor sleep in the relation between workplace bullying/unwanted sexual attention and long-term sickness absence. Int Arch Occup Environ Health. 2016;89(6):967-79.

40. Ndumele CE, Matsushita K, Lazo M, Bello N, Blumenthal RS, Gerstenblith G, et al. Obesity and Subtypes of Incident Cardiovascular Disease. J Am Heart Assoc. 2016;5(8).

41. O'Reilly K, O'Connell P, Donohoe G, Coyle C, O'Sullivan D, Azvee Z, et al. Anticholinergic burden in schizophrenia and ability to benefit from psychosocial treatment programmes: a 3-year prospective cohort study. Psychol Med. 2016;46(15):3199-211.

42. Patel R, Wilson R, Jackson R, Ball M, Shetty H, Broadbent M, et al. Association of cannabis use with hospital admission and antipsychotic treatment failure in first episode psychosis: An observational study. BMJ Open. 2016;6(3).

43. Pechey R, Monsivais P. Socioeconomic inequalities in the healthiness of food choices: Exploring the contributions of food expenditures. Prev Med. 2016;88:203-9.

44. Putrik P, Ramiro S, Keszei AP, Hmamouchi I, Dougados M, Uhlig T, et al. Lower education and living in countries with lower wealth are associated with higher disease activity in rheumatoid arthritis: results from the multinational COMORA study. Ann Rheum Dis. 2016;75(3):540-6.

45. Rode JL, Kiel EJ. The mediated effects of maternal depression and infant temperament on maternal role. Arch Womens Ment Health. 2016;19(1):133-40.

46. Siviero P, Zambon S, Limongi F, Castell MV, Cooper C, Deeg DJ, et al. How Hand Osteoarthritis, Comorbidity, and Pain Interact to Determine Functional Limitation in Older People: Observations From the European Project on OSteoArthritis Study. Arthritis Rheumatol. 2016;68(11):2662-70.

47. Spauwen PJ, Martens RJ, Stehouwer CD, Verhey FR, Schram MT, Sep SJ, et al. Lower verbal intelligence is associated with diabetic complications and slower walking speed in people with Type 2 diabetes: the Maastricht Study. Diabet Med. 2016;33(12):1632-9.

48. Tung YC, Chang GM. The Relationships Among Regionalization, Processes, and Outcomes for Stroke Care: A Nationwide Population-based Study. Medicine (Baltimore). 2016;95(15):e3327.

49. Van Allen J, Steele RG, Nelson MB, Peugh J, Egan A, Clements M, et al. A Longitudinal Examination of Hope and Optimism and Their Role in Type 1 Diabetes in Youths. J Pediatr Psychol. 2016;41(7):741-9.

50. Wu JR, Lennie TA, Frazier SK, Moser DK. Health-Related Quality of Life, Functional Status, and Cardiac Event-Free Survival in Patients With Heart Failure. J Cardiovasc Nurs. 2016;31(3):236-44.

51. Afshar M, Netzer G, Mosier MJ, Cooper RS, Adams W, Burnham EL, et al. The Contributing Risk of Tobacco Use for ARDS Development in Burn-Injured Adults With Inhalation Injury. Respir Care. 2017;62(11):1456-65.

52. Ananth CV, Friedman AM, Lavery JA, VanderWeele TJ, Keim S, Williams MA. Neurodevelopmental outcomes in children in relation to placental abruption. Bjog. 2017;124(3):463-72.

53. Bakker RM, Kenter GG, Creutzberg CL, Stiggelbout AM, Derks M, Mingelen W, et al. Sexual distress and associated factors among cervical cancer survivors: A cross-sectional multicenter observational study. Psycho-oncology. 2017;26(10):1470-7.

54. Banas K, Lyimo RA, Hospers HJ, van der Ven A, de Bruin M. Predicting adherence to combination antiretroviral therapy for HIV in Tanzania: A test of an extended theory of planned behaviour model. Psychol Health. 2017;32(10):1249-65.

55. Bouwmans MEJ, Bos EH, Hoenders HJR, Oldehinkel AJ, de Jonge P. Sleep quality predicts positive and negative affect but not vice versa. An electronic diary study in depressed and healthy individuals. J Affect Disord. 2017;207:260-7.

56. Brown J, Coxon K, Fong C, Clarke E, Rogers K, Keay L. Seat belt repositioning and use of vehicle seat cushions is increased among older drivers aged 75 years and older with morbidities. Australas J Ageing. 2017;36(1):26-31.

57. Bulls HW, Lynch MK, Petrov ME, Gossett EW, Owens MA, Terry SC, et al. Depressive Symptoms and Sleep Efficiency Sequentially Mediate Racial Differences in Temporal Summation of Mechanical Pain. Ann Behav Med. 2017;51(5):673-82.

58. Chen E, Shalowitz MU, Story RE, Ehrlich KB, Manczak EM, Ham PJ, et al. Parents' childhood socioeconomic circumstances are associated with their children's asthma outcomes. J Allergy Clin Immunol. 2017;140(3):828-35.e2.

59. Cook RL, Zhou Z, Kelso-Chichetto NE, Janelle J, Morano JP, Somboonwit C, et al. Alcohol consumption patterns and HIV viral suppression among persons receiving HIV care in Florida: an observational study. Addict Sci Clin Pract. 2017;12(1):22.

60. Fritz J, Shiffman D, Melander O, Tada H, Ulmer H. Metabolic Mediators of the Effects of Family History and Genetic Risk Score on Coronary Heart Disease-Findings From the Malmö Diet and Cancer Study. J Am Heart Assoc. 2017;6(3).

61. Garre-Olmo J, Vilalta-Franch J, Calvó-Perxas L, López-Pousa S. A Path Analysis of Dependence and Quality of Life in Alzheimer's Disease. Am J of Alzheimer's Dis Other Dement. 2017;32(2):108-15.

62. Gillespie SL, Christian LM, Alston AD, Salsberry PJ. Childhood stress and birth timing among African American women: Cortisol as biological mediator. Psychoneuroendocrinology. 2017;84:32-41.

63. Giummarra MJ, Baker KS, Ioannou L, Gwini SM, Gibson SJ, Arnold CA, et al. Associations between compensable injury, perceived fault and pain and disability 1 year after injury: A registry-based Australian cohort study. BMJ Open. 2017;7(10).

64. Hendriks SA, Smalbrugge M, van Gageldonk-Lafeber AB, Galindo-Garre F, Schipper M, Hertogh C, et al. Pneumonia, Intake Problems, and Survival Among Nursing Home Residents With Variable Stages of Dementia in the Netherlands: Results From a Prospective Observational Study. Alzheimer Dis Assoc Disord. 2017;31(3):200-8.

65. Hodge DR, Zidan T, Husain A. Examining the relationship between encouragement and health-related quality of life among Muslims. Soc Work Health Care. 2017;56(6):470-87.

66. Jung SY, Barrington WE, Lane DS, Chen C, Chlebowski R, Corbie-Smith G, et al. Bioavailable insulin-like growth factor-I as mediator of racial disparity in obesity-relevant breast and colorectal cancer risk among postmenopausal women. Menopause. 2017;24(3):288-98.

67. Koelmel E, Hughes AJ, Alschuler KN, Ehde DM. Resilience Mediates the Longitudinal Relationships Between Social Support and Mental Health Outcomes in Multiple Sclerosis. Arch Phys Med Rehabil. 2017;98(6):1139-48.

68. Lau EY, Saunders RP, Beets MW, Cai B, Pate RR. Factors influencing implementation of a preschool-based physical activity intervention. Health Educ Res. 2017;32(1):69-80.

69. McHugh Power JE, Lawlor BA, Kee F. Social support mediates the relationships between extraversion, neuroticism, and cognitive function in older adults. Public Health. 2017;147:144-52.

70. Mefford M, Safford MM, Muntner P, Durant RW, Brown TM, Levitan EB. Insurance, self-reported medication adherence and LDL cholesterol: The REasons for Geographic And Racial Differences in Stroke study. Int J Cardiol. 2017;236:462-5.

71. Miljanović M, Sindik J, Milunović V, Kralj Škoc V, Braš M, Đorđević V, et al. Psychosocial Determinants of Satisfaction with Hospital Care in Adult Patients Suffering from Advanced Cancer. Acta Clin Croat. 2017;56(2):218-26.

72. Park KH, Kim CY, Cha J, Kim Y, Lee J, Choi J. A path analysis of effects of patients' underlying conditions, treatment satisfaction and adherence on quality of life among Korea glaucoma patients: Results from Korea glaucoma outcomes research. Value Health. 2017;20(9):A805.

73. Pedersen JM, Budtz-Jørgensen E, De Roos A, Garcia L, Lund R, Rod NH, et al. Understanding the relation between socioeconomic position and inflammation in post-menopausal women: education, income and occupational prestige. Eur J Public Health. 2017;27(6):1074-9.

74. Potthoff S, Presseau J, Sniehotta FF, Johnston M, Elovainio M, Avery L. Planning to be routine: habit as a mediator of the planning-behaviour relationship in healthcare professionals. Implement Sci. 2017;12(1):24.

75. Schulz P, Beblo T, Ribbert H, Kater L, Spannhorst S, Driessen M, et al. How is childhood emotional abuse related to major depression in adulthood? The role of personality and emotion acceptance. Child Abuse Negl. 2017;72:98-109.

76. Smarius LJ, Strieder TG, Loomans EM, Doreleijers TA, Vrijkotte TG, Gemke RJ, et al. Excessive infant crying doubles the risk of mood and behavioral problems at age 5: evidence for mediation by maternal characteristics. Eur Child Adolesc Psychiatry. 2017;26(3):293-302.

77. Whitworth JW, Craft LL, Dunsiger SI, Ciccolo JT. Direct and indirect effects of exercise on posttraumatic stress disorder symptoms: A longitudinal study. Gen Hosp Psychiatry. 2017;49:56-62.

78. Young DA, Shumway M, Flentje A, Riley ED. The relationship between childhood abuse and violent victimization in homeless and marginally housed women: The role of dissociation as a potential mediator. Psychol Trauma. 2017;9(5):613-21.

79. Alhalal E, Ford-Gilboe M, Wong C, Albuhairan F. Factors mediating the impacts of child abuse and intimate partner violence on chronic pain: A cross-sectional study 11 Medical and Health Sciences 1117 Public Health and Health Services 17 Psychology and Cognitive Sciences 1701 Psychology. BMC Women's Health. 2018;18(1).

80. Assmann KE, Ruhunuhewa I, Adjibade M, Li Z, Varraso R, Hercberg S, et al. The Mediating Role of Overweight and Obesity in the Prospective Association between Overall Dietary Quality and Healthy Aging. Nutrients. 2018;10(4).

81. Bagshaw SM, Wang X, Zygun DA, Zuege D, Dodek P, Garland A, et al. Association between strained capacity and mortality among patients admitted to intensive care: A path-analysis modeling strategy. J Crit Care. 2018;43:81-7.

82. Betka S, Pfeifer G, Garfinkel S, Prins H, Bond R, Sequeira H, et al. How Do Self-Assessment of Alexithymia and Sensitivity to Bodily Sensations Relate to Alcohol Consumption? Alcohol Clin Exp Res. 2018;42(1):81-8.

83. Broström A, Wahlin A, Alehagen U, Ulander M, Johansson P. Sex-Specific Associations Between Self-reported Sleep Duration, Cardiovascular Disease, Hypertension, and Mortality in an Elderly Population. J Cardiovasc Nurs. 2018;33(5):422-8.

84. Carmona NE, Subramaniapillai M, Mansur RB, Cha DS, Lee Y, Fus D, et al. Sex differences in the mediators of functional disability in Major Depressive Disorder. J Psychiatr Res. 2018;96:108-14.

85. Düzel E, Berron D, Schütze H, Cardenas-Blanco A, Metzger C, Betts M, et al. CSF total tau levels are associated with hippocampal novelty irrespective of hippocampal volume. Alzheimers Dement (Amst). 2018;10:782-90.

86. Dworkin ER, Gilmore AK, Bedard-Gilligan M, Lehavot K, Guttmannova K, Kaysen D. Predicting PTSD severity from experiences of trauma and heterosexism in lesbian and bisexual women: A longitudinal study of cognitive mediators. J Couns Psychol. 2018;65(3):324-33.

87. Falck RS, Best JR, Drenowatz C, Hand GA, Shook RP, Lavie CJ, et al. Psychosocial Determinants of Weight Loss Among Young Adults With Overweight and Obesity: How Does Drive for Thinness Affect Weight Loss? J Cardiopulm Rehabil Prev. 2018;38(2):104-10.

88. Gillespie SL, Mitchell AM, Kowalsky JM, Christian LM. Maternal parity and perinatal cortisol adaptation: The role of pregnancy-specific distress and implications for postpartum mood. Psychoneuroendocrinology. 2018;97:86-93.

89. Henneghan A, Stuifbergen A, Becker H, Kesler S, King E. Modifiable correlates of perceived cognitive function in breast cancer survivors up to 10 years after chemotherapy completion. J Cancer Surviv. 2018;12(2):224-33.

90. Holbein CE, Fogleman ND, Hommel K, Apers S, Rassart J, Moons P, et al. A multinational observational investigation of illness perceptions and quality of life among patients with a Fontan circulation. Congenit Heart Dis. 2018;13(3):392-400.

91. Hurd NM, Albright J, Wittrup A, Negrete A, Billingsley J. Appraisal Support from Natural Mentors, Self-worth, and Psychological Distress: Examining the Experiences of Underrepresented Students Transitioning Through College. J Youth Adolesc. 2018;47(5):1100-12.

92. Jang BJ, Schuler MS, Evans-Polce RJ, Patrick ME. Marital Status as a Partial Mediator of the Associations Between Young Adult Substance Use and Subsequent Substance Use Disorder: Application of Causal Inference Methods. J Stud Alcohol Drugs. 2018;79(4):567-77.

93. Jones MC, Smith K, Herber O, White M, Steele F, Johnston DW. Intention, beliefs and mood assessed using electronic diaries predicts attendance at cardiac rehabilitation: An observational study. Int J Nurs Stud. 2018;88:143-52.

94. Khan A, Teoh KR, Islam S, Hassard J. Psychosocial work characteristics, burnout, psychological morbidity symptoms and early retirement intentions: a cross-sectional study of NHS consultants in the UK. BMJ Open. 2018;8(7):e018720.

95. Lamarche F, Agharazii M, Nadeau-Fredette AC, Madore F, Goupil R. Central and Brachial Blood Pressures, Statins, and Low-Density Lipoprotein Cholesterol: A Mediation Analysis. Hypertension. 2018;71(3):415-21.

96. Lange D, Corbett J, Knoll N, Schwarzer R, Lippke S. Fruit and Vegetable Intake: the Interplay of Planning, Social Support, and Sex. Int J Behav Med. 2018;25(4):421-30.

97. Lee MR, Jung SM, Bang H, Kim HS, Kim YB. Association between muscle strength and type 2 diabetes mellitus in adults in Korea: Data from the Korea national health and nutrition examination survey (KNHANES) VI. Medicine (Baltimore). 2018;97(23):e10984.

98. Li Y, Zhang T, Han T, Li S, Bazzano L, He J, et al. Impact of cigarette smoking on the relationship between body mass index and insulin: Longitudinal observation from the Bogalusa Heart Study. Diabetes Obes Metab. 2018;20(7):1578-84.

99. Low S, Zhang X, Wang J, Yeoh LY, Liu YL, Ang KKL, et al. Long-term prospective observation suggests that glomerular hyperfiltration is associated with rapid decline in renal filtration function: A multiethnic study. Diab Vasc Dis Res. 2018;15(5):417-23.

100. MacKinnon AL, Carter CS, Feeley N, Gold I, Hayton B, Santhakumaran S, et al. Theory of mind as a link between oxytocin and maternal behavior. Psychoneuroendocrinology. 2018;92:87-94.

101. Malivoire BL, Hare CJ, Hart TL. Psychological symptoms and perceived cognitive impairment in multiple sclerosis: The role of rumination. Rehabil Psychol. 2018;63(2):286-94.

102. Meli L, Kautz M, Julian J, Edmondson D, Sumner JA. The role of perceived threat during emergency department cardiac evaluation and the age-posttraumatic stress disorder link. J Behav Med. 2018;41(3):357-63.

103. Owari Y, Miyatake N. Relationship between Chronic Low Back Pain, Social Participation, and Psychological Distress in Elderly People : A Pilot Mediation Analysis. Acta Med Okayama. 2018;72(4):337-42.

104. Pedersen M, Egerod I, Overgaard D, Baastrup M, Andersen I. Social inequality in phase II cardiac rehabilitation attendance: The impact of potential mediators. Eur J Cardiovasc Nurs. 2018;17(4):345-55.

105. Peeters G, Leahy S, Kennelly S, Kenny RA. Is Fear of Falling Associated With Decline in Global Cognitive Functioning in Older Adults: Findings From the Irish Longitudinal Study on Ageing. J Am Med Dir Assoc. 2018;19(3):248-54.e3.

106. Peng H, Zhu Y, Strachan E, Fowler E, Bacus T, Roy-Byrne P, et al. Childhood Trauma, DNA Methylation of Stress-Related Genes, and Depression: Findings From Two Monozygotic Twin Studies. Psychosom Med. 2018;80(7):599-608.

107. Rathbun AM, Shardell MD, Stuart EA, Yau MS, Gallo JJ, Schuler MS, et al. Pain severity as a mediator of the association between depressive symptoms and physical performance in knee osteoarthritis. Osteoarthr Cartil. 2018;26(11):1453-60.

108. Reed-Knight B, van Tilburg MAL, Levy RL, Langer SL, Romano JM, Murphy TB, et al. Maladaptive Coping and Depressive Symptoms Partially Explain the Association Between Family Stress and Pain-Related Distress in Youth With IBD. J Pediatr Psychol. 2018;43(1):94-103.

109. Reeve S, Nickless A, Sheaves B, Freeman D. Insomnia, negative affect, and psychotic experiences: Modelling pathways over time in a clinical observational study. Psychiatry Res. 2018;269:673-80.

110. Reilly JP, Wang F, Jones TK, Palakshappa JA, Anderson BJ, Shashaty MGS, et al. Plasma angiopoietin-2 as a potential causal marker in sepsis-associated ARDS development: evidence from Mendelian randomization and mediation analysis. Intensive Care Med. 2018;44(11):1849-58.

111. Richter MS, O'Reilly K, O'Sullivan D, O'Flynn P, Corvin A, Donohoe G, et al. Prospective observational cohort study of 'treatment as usual' over four years for patients with schizophrenia in a national forensic hospital. BMC Psychiatry. 2018;18(1):289.

112. Schrepf A, Naliboff B, Williams DA, Stephens-Shields AJ, Landis JR, Gupta A, et al. Adverse Childhood Experiences and Symptoms of Urologic Chronic Pelvic Pain Syndrome: A Multidisciplinary Approach to the Study of Chronic Pelvic Pain Research Network Study. Ann Behav Med. 2018;52(10):865-77.

113. Seixas AA, Vallon J, Barnes-Grant A, Butler M, Langford AT, Grandner MA, et al. Mediating effects of body mass index, physical activity, and emotional distress on the relationship between short sleep and cardiovascular disease. Medicine (Baltimore). 2018;97(37):e11939.

114. Seng EK, Kuka AJ, Mayson SJ, Smitherman TA, Buse DC. Acceptance, Psychiatric Symptoms, and Migraine Disability: An Observational Study in a Headache Center. Headache. 2018;58(6):859-72.

115. Shao B, Song B, Feng S, Lin Y, Du J, Shao H, et al. The relationship of social support, mental health, and health-related quality of life in human immunodeficiency virus-positive men who have sex with men: From the analysis of canonical correlation and structural equation model: A cross-sectional study. Medicine (Baltimore). 2018;97(30):e11652.

116. Shippee ND, Finch M, Wholey DR. Assessing medical home mechanisms: Certification, asthma education, and outcomes. Am J Manag Care. 2018;24(3):e79-e85.

117. Skakkebæk A, Moore PJ, Pedersen AD, Bojesen A, Kristensen MK, Fedder J, et al. Anxiety and depression in Klinefelter syndrome: The impact of personality and social engagement. PLoS One. 2018;13(11):e0206932.

118. Smit K, Otten R, Voogt C, Kleinjan M, Engels R, Kuntsche E. Exposure to drinking mediates the association between parental alcohol use and preteen alcohol use. Addictive Behaviors. 2018;87:244-50.

119. Song EK, Wu JR. Associations of Vitamin D Intake and Sleep Quality With Cognitive Dysfunction in Older Adults With Heart Failure. J Cardiovasc Nurs. 2018;33(4):392-9.

120. Swinnen TW, Vlaeyen JWS, Dankaerts W, Westhovens R, de Vlam K. Activity Limitations in Patients with Axial Spondyloarthritis: A Role for Fear of Movement and (Re)injury Beliefs. J Rheumatol. 2018;45(3):357-66.

121. Talaei-Khoei M, Chen N, Ring D, Vranceanu AM. Satisfaction with life moderates the indirect effect of pain intensity on pain interference through pain catastrophizing. J Consult Clin Psychol. 2018;86(3):231-41.

122. Talbot D, Delaney JAC, Sandfort V, Herrington DM, McClelland RL. Importance of the lipid-related pathways in the association between statins, mortality, and cardiovascular disease risk: The Multi-Ethnic Study of Atherosclerosis. Pharmacoepidemiol Drug Saf. 2018;27(4):365-72.

123. Tun HM, Bridgman SL, Chari R, Field CJ, Guttman DS, Becker AB, et al. Roles of Birth Mode and Infant Gut Microbiota in Intergenerational Transmission of Overweight and Obesity From Mother to Offspring. JAMA Pediatr. 2018;172(4):368-77.

124. Wagner NJ, Gueron-Sela N, Bedford R, Propper C. Maternal Attributions of Infant Behavior and Parenting in Toddlerhood Predict Teacher-Rated Internalizing Problems in Childhood. J Clin Child Adolesc Psychol. 2018;47:S569-s77.

125. Albelda R, Wiemers E, Hahn T, Khera N, Salas Coronado DY, Abel GA. Relationship between paid leave, financial burden, and patient-reported outcomes among employed patients who have undergone bone marrow transplantation. Qual Life Res. 2019;28(7):1835-47.

126. Albritton JA, Fried B, Singh K, Weiner BJ, Reeve B, Edwards JR. The role of psychological safety and learning behavior in the development of effective quality improvement teams in Ghana: an observational study. BMC Health Serv Res. 2019;19(1):385.

127. Bollyky TJ, Templin T, Cohen M, Schoder D, Dieleman JL, Wigley S. The relationships between democratic experience, adult health, and cause-specific mortality in 170 countries between 1980 and 2016: an observational analysis. The Lancet. 2019;393(10181):1628-40.

128. Caldwell JA, Knapik JJ, Shing TL, Kardouni JR, Lieberman HR. The association of insomnia and sleep apnea with deployment and combat exposure in the entire population of US army soldiers from 1997 to 2011: a retrospective cohort investigation. Sleep. 2019;42(8).

129. Chang KC, Lin CY, Chang CC, Ting SY, Cheng CM, Wang JD. Psychological distress mediated the effects of self-stigma on quality of life in opioid-dependent individuals: A cross-sectional study. PLoS One. 2019;14(2):e0211033.

130. Choe YR, Jeong JR, Kim YP. Grip strength mediates the relationship between muscle mass and frailty. J Cachexia Sarcopeni. 2019.

131. Cui Y, Kim SW, Lee BJ, Kim JJ, Yu JC, Lee KY, et al. Negative schema and rumination as mediators of the relationship between childhood trauma and recent suicidal ideation in patients with early psychosis. J Clin Psychiat. 2019;80(3).

132. De La Fuente J, García-Torrecillas JM, Solinas G, Iglesias-Espinosa MM, Garzón-Umerenkova A, Fiz-Pérez J. Structural equation model (SEM) of stroke mortality in Spanish inpatient hospital settings: The role of individual and contextual factors. Front Neurol. 2019;10.

133. De Oliveira Neves FM, Araújo CB, De Freitas DF, Arruda BFT, De Macêdo Filho LJM, Salles VB, et al. Fibroblast growth factor 23, endothelium biomarkers and acute kidney injury in critically-ill patients. J Transl Med. 2019;17(1).

134. Eisenga MF, De Jong MA, Van der Meer P, Leaf DE, Huls G, Nolte IM, et al. Iron deficiency, elevated erythropoietin, fibroblast growth factor 23, and mortality in the general population of the netherlands: A cohort study. PLoS Medicine. 2019;16(6).

135. Fujita Y, Kouda K, Ohara K, Nakamura H, Iki M. Leptin mediates the relationship between fat mass and blood pressure: The Hamamatsu School-based health study. Medicine (Baltimore). 2019;98(12):e14934.

136. Garrido-Miguel M, Torres-Costoso A, Martínez-Andrés M, Notario-Pacheco B, Díez-Fernández A, Álvarez-Bueno C, et al. The risk of eating disorders and bone health in young adults: the mediating role of body composition and fitness. Eat Weight Disord. 2019;24(6):1145-54.

137. Gentina T, Bailly S, Jounieaux F, Verkindre C, Broussier PM, Guffroy D, et al. Marital quality, partner's engagement and continuous positive airway pressure adherence in obstructive sleep apnea. Sleep Med. 2019;55:56-61.

138. Guadamuz JS, Ozenberger K, Qato DM, Ko NY, Saffore CD, Adimadhyam S, et al. Mediation analyses of socioeconomic factors determining racial differences in the treatment of diffuse large B-cell lymphoma in a cohort of older adults. Medicine (Baltimore). 2019;98(46):e17960.

139. Huang X, Li Z, Wan Q. From organisational justice to turnover intention among community nurses: A mediating model. J Clin Nurs. 2019;28(21):3957-65.

140. Jordahl KM, Phipps AI, Randolph TW, Tindle HA, Liu S, Tinker LF, et al. Differential DNA methylation in blood as a mediator of the association between cigarette smoking and bladder cancer risk among postmenopausal women. Epigenetics. 2019;14(11):1065-73.

141. Kasten S, van Osch L, Candel M, de Vries H. The influence of pre-motivational factors on behavior via motivational factors: a test of the I-Change model. BMC Psychol. 2019;7(1):7.

142. Kelley ML, Bravo AJ, Hamrick HC, Braitman AL, Judah MR. Killing during combat and negative mental health and substance use outcomes among recent-era veterans: The mediating effects of rumination. Psychol Trauma. 2019;11(4):379-82.

143. Lertpimonchai A, Rattanasiri S, Tamsailom S, Champaiboon C, Ingsathit A, Kitiyakara C, et al. Periodontitis as the risk factor of chronic kidney disease: Mediation analysis. J Clin Periodontol. 2019;46(6):631-9.

144. Li HC, Chen KM, Hsu HF. Modelling factors of urinary incontinence in institutional older adults with dementia. J Clin Nurs. 2019;28(23):4504-12.

145. Li X, Qian Y, Xu H, Guan J, Yin S. Interrelationships among common predictors of cardiovascular diseases in patients of OSA: a large-scale observational study. Sleep Med. 2019;64:S310.

146. Lindner OC, McCabe MG, Boele F, Mayes A, Talmi D, Radford J, et al. Discussing factors associated with quality of life in cancer follow-up appointments: a preliminary test of a pragmatic model for clinical practice. Clin Rehabil. 2019;33(4):762-72.

147. Loskutova N, Watts AS, Burns JM. The cause-effect relationship between bone loss and Alzheimer's disease using statistical modeling. Med Hypotheses. 2019;122:92-7.

148. Loyd BJ, Stackhouse SK, Hogan C, Dayton MR, Stevens-Lapsley JE, Kittelson AJ. Peripheral Nociception Is Associated with Voluntary Activation Deficits and Quadriceps Weakness Following Total Knee Arthroplasty. J Bone Joint Surg Am. 2019;101(17):1539-45.

149. Margiotta DPE, Fasano S, Basta F, Pierro L, Riccardi A, Navarini L, et al. The association between duration of remission, fatigue, depression and health-related quality of life in Italian patients with systemic lupus erythematosus. Lupus. 2019;28(14):1705-11.

150. Matenchuk BA, Tamana SK, Lou WYW, Lefebvre DL, Sears MR, Becker AB, et al. Prenatal depression and birth mode sequentially mediate maternal education's influence on infant sleep duration. Sleep Med. 2019;59:24-32.

151. Matsuishi Y, Shimojo N, Unoki T, Sakuramoto H, Tokunaga C, Yoshino Y, et al. Type D personality is a predictor of prolonged acute brain dysfunction (delirium/coma) after cardiovascular surgery. BMC Psychol. 2019;7(1):27.

152. Matthews KA, Hall MH, Lee L, Kravitz HM, Chang Y, Appelhans BM, et al. Racial/ethnic disparities in women's sleep duration, continuity, and quality, and their statistical mediators: Study of women's health across the nation. Sleep. 2019;42(5).

153. Miller MB, Metrik J, Borsari B, Jackson KM. Longitudinal Associations between Sleep, Intrusive Thoughts, and Alcohol Problems Among Veterans. Alcohol Clin Exp Res. 2019;43(11):2438-45.

154. Müller A, Znoj H, Moggi F. How Are Self-Efficacy and Motivation Related to Drinking Five Years after Residential Treatment? A Longitudinal Multicenter Study. Eur Addict Res. 2019;25(5):213-23.

155. Oliveira DVD, Nascimento Júnior JRAD, Cunha PMD, Cavaglieri CR. Relationship between the practice of physical activity and the functionality of older people from primary care health: Mediation by sedentary behavior. Apunts Medicina de l'Esport. 2019;54(202):45-53.

156. Opel N, Redlich R, Dohm K, Zaremba D, Goltermann J, Repple J, et al. Mediation of the influence of childhood maltreatment on depression relapse by cortical structure: a 2-year longitudinal observational study. Lancet Psychiat. 2019;6(4):318-26.

157. Ower C, Kemmler G, Vill T, Martini C, Schmitt A, Sperner-Unterweger B, et al. The effect of physical activity in an alpine environment on quality of life is mediated by resilience in patients with psychosomatic disorders and healthy controls. Eur Arch Psy Clin Neurosci. 2019;269(5):543-53.

158. Palese A, Grassetti L, Bressan V, Decaro A, Kasa T, Longobardi M, et al. A path analysis on the direct and indirect effects of the unit environment on eating dependence among cognitively impaired nursing home residents. BMC Health Serv Res. 2019;19(1):775.

159. Papaioannou TG, Oikonomou E, Lazaros G, Christoforatou E, Vogiatzi G, Tsalamandris S, et al. The influence of resting heart rate on pulse wave velocity measurement is mediated by blood pressure and depends on aortic stiffness levels: insights from the Corinthia study. Physiol Meas. 2019;40(5):055005.

160. Parpa E, Kostopoulou S, Tsilika E, Galanos A, Mystakidou K. Depression as a Mediator or Moderator Between Preparatory Grief and Sense of Dignity in Patients With Advanced Cancer. Am J Hosp Palliat Care. 2019;36(12):1063-7.

161. Progovac AM, Pettinger M, Donohue JM, Chang CCHJ, Matthews KA, Habermann EB, et al. Optimism may moderate screening mammogram frequency in Medicare: A longitudinal study. Medicine (United States). 2019;98(24).

162. Putrik P, Ramiro S, Moltó A, Keszei AP, Norton S, Dougados M, et al. Individual-level and country-level socioeconomic determinants of disease outcomes in SpA: Multinational, cross-sectional study (ASAS-COMOSPA). Ann Rheum Dis. 2019;78(4):486-93.

163. Rogante E, Sarubbi S, Lamis DA, Canzonetta V, Sparagna A, De Angelis V, et al. Illness Perception and Job Satisfaction in Patients Suffering from Migraine Headaches: Trait Anxiety and Depressive Symptoms as Potential Mediators. Headache. 2019;59(1):46-55.

164. Romano L, Buonomo I, Callea A, Fiorilli C. Alexithymia in Young people's academic career: The mediating role of anxiety and resilience. J Genet Psychol. 2019;180(4):157-69.

165. Rothenberg KA, Stern JR, George EL, Trickey AW, Morris AM, Hall DE, et al. Association of Frailty and Postoperative Complications With Unplanned Readmissions After Elective Outpatient Surgery. JAMA Netw Open. 2019;2(5):e194330.

166. Schwartz CE, Zhang J, Stucky BD, Michael W, Rapkin BD. Is the link between socioeconomic status and resilience mediated by reserve-building activities: mediation analysis of web-based cross-sectional data from chronic medical illness patient panels. BMJ Open. 2019;9(5):e025602.

167. Song J, Jiang X, Juan J, Cao Y, Chibnik LB, Hofman A, et al. Role of metabolic syndrome and its components as mediators of the genetic effect on type 2 diabetes: A family-based study in China. J Diabetes. 2019;11(7):552-62.

168. Su H, Wang L, Li Y, Yu H, Zhang J. The mediating and moderating roles of self-acceptance and self-reported health in the relationship between self-worth and subjective well-being among elderly Chinese rural empty-nester: An observational study. Medicine (Baltimore). 2019;98(28):e16149.

169. Tawakol A, Osborne MT, Wang Y, Hammed B, Tung B, Patrich T, et al. Stress-Associated Neurobiological Pathway Linking Socioeconomic Disparities to Cardiovascular Disease. J Am Coll Cardiol. 2019;73(25):3243-55.

170. Turan B, Rice WS, Crockett KB, Johnson M, Neilands TB, Ross SN, et al. Longitudinal association between internalized HIV stigma and antiretroviral therapy adherence for women living with HIV: the mediating role of depression. Aids. 2019;33(3):571-6.

171. Tutek J, Albright AE, Lichstein KL. Nonrestorative sleep mediates eveningness and insomnia severity. Sleep Biol Rhythms. 2019;17(1):73-8.

172. Yang S, Huang S, Daniels LB, Yeboah J, Lima JAC, Cannone V, et al. NT-proBNP, race and endothelial function in the Multi-Ethnic Study of Atherosclerosis. Heart. 2019;105(20):1590-6.

173. Yeh VM, Mayberry LS, Bachmann JM, Wallston KA, Roumie C, Muñoz D, et al. Depressed Mood, Perceived Health Competence and Health Behaviors: aCross-Sectional Mediation Study in Outpatients with Coronary Heart Disease. J Gen Intern Med. 2019;34(7):1123-30.

174. Zhang CJP, Barnett A, Sit CHP, Lai PC, Johnston JM, Lee RSY, et al. To what extent does physical activity explain the associations between neighborhood environment and depressive symptoms in older adults living in an Asian metropolis? Ment Health Phys Act. 2019;16:96-104.
