# Supplementary material for: Mediation analysis methods used in observational research: a scoping review and recommendations
Source: BMC Med Res Methodol. 2021 Oct 25;21:226. doi: 10.1186/s12874-021-01426-3 (PMC8543973; doi:10.1186/s12874-021-01426-3)
Supplement: Supplementary file 5 — Additional file 5: Supplementary appendix 5. Overview of mediation analysis methods used to analyze repeated measurements in the included papers. [file 12874_2021_1426_MOESM5_ESM.docx]

**Supplementary appendix 5: Overview of mediation analysis methods used to analyze repeated measurements in the included papers**

| **Table S1.** Methods Used to Estimate Mediation Models Based on Repeated Measurements (*n*=41). | |
| --- | --- |
| **Method** | ***n* (%)** |
| Adjustment for measurement at the first time point | 6 (14.6) |
| Change scores | 6 (14.6) |
| Change scores and adjustment for measurement at the first time point | 3 (7.3) |
| Computation of a variable that indicates increase or decline over time | 2 (4.9) |
| Computation a variable that indicates increase or decline over time and adjustment for measurement at the first time point | 1 (2.4) |
| Cross-lagged panel model | 2 (4.9) |
| Exposure and outcome are the same variable, but measured at different time points | 2 (4.9) |
| Latent growth-curve model | 1 (2.4) |
| Based on slopes from a latent growth-curve model | 1 (2.4) |
| Multilevel models with the participant as the cluster level | 4 (9.8) |
| Generalized estimation equations (GEE) | 1 (2.4) |
| Autoregressive integrated moving average model (ARIMA) | 1 (2.4) |
| Joint models and Cox proportional hazards models with time-dependent variables | 1 (2.4) |
| Computation of a latent variable based on repeated measures | 1 (2.4) |
| Estimation based on person years | 1 (2.4) |
| Stratified by time point | 5 (12.2) |
| Unclear (repeated measures were mentioned, but the used methodology for repeated measures mediation analysis remained unclear). | 3 (7.3) |
